# Exciton Delocalization Counteracts the Energy Gap: A New Pathway toward NIR-Emissive Dyes

**DOI:** 10.1021/jacs.1c10654

**Published:** 2021-11-08

**Authors:** Alexei Cravcenco, Yi Yu, Fredrik Edhborg, Jonas F. Goebel, Zoltan Takacs, Yizhou Yang, Bo Albinsson, Karl Börjesson

**Affiliations:** † Department of Chemistry and Molecular Biology, University of Gothenburg, Kemivägen 10, 41296 Gothenburg, Sweden; ‡ Department of Chemistry and Chemical Engineering, Chalmers University of Technology, Kemivägen 10, 41296 Gothenburg, Sweden; § Swedish NMR Centre, University of Gothenburg, Medicinaregatan 5C, 40530 Gothenburg, Sweden

## Abstract

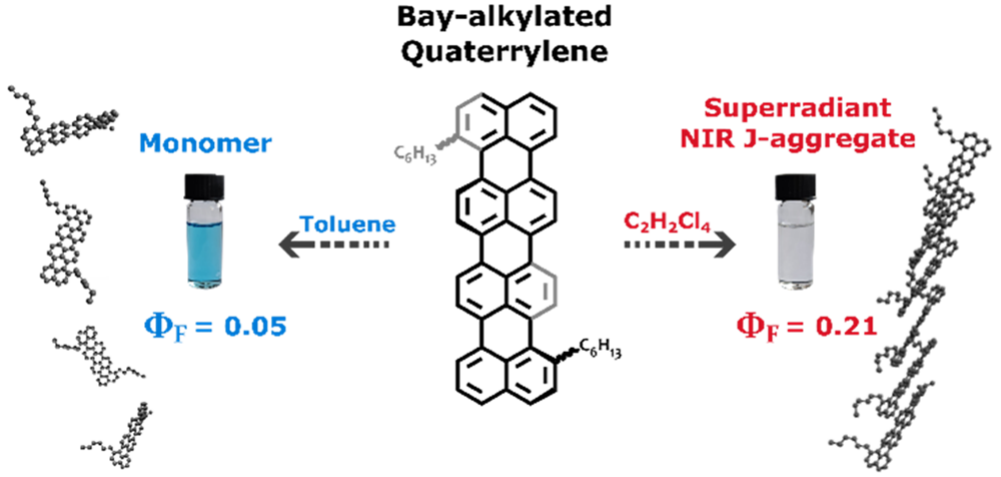

Exciton coupling between the transition dipole moments of ordered dyes in supramolecular assemblies, so-called J/H-aggregates, leads to shifted electronic transitions. This can lower the excited state energy, allowing for emission well into the near-infrared regime. However, as we show here, it is not only the excited state energy modifications that J-aggregates can provide. A bay-alkylated quaterrylene was synthesized, which was found to form J-aggregates in 1,1,2,2-tetrachloroethane. A combination of superradiance and a decreased nonradiative relaxation rate made the J-aggregate four times more emissive than the monomeric counterpart. A reduced nonradiative relaxation rate is a nonintuitive consequence following the 180 nm (3300 cm^–1^) red-shift of the J-aggregate in comparison to the monomeric absorption. However, the energy gap law, which is commonly invoked to rationalize increased nonradiative relaxation rates with increasing emission wavelength, also contains a reorganization energy term. The reorganization energy is highly suppressed in J-aggregates due to exciton delocalization, and the framework of the energy gap law could therefore reproduce our experimental observations. J-Aggregates can thus circumvent the common belief that lowering the excited state energies results in large nonradiative relaxation rates and are thus a pathway toward highly emissive organic dyes in the NIR regime.

## Introduction

Near-infrared (NIR) emissive organic dyes are of importance in applications such as organic light-emitting diodes (OLEDs),^1^ bioimaging^2^ photodetection,^3^ and solar energy harvesting.^4^ However, designing organic dyes having efficient emission in the near-IR regime is difficult for mainly two reasons. First, the very low energy electronic transition required to emit a photon in this regime enforces a conjugated system of considerable size. This makes synthesis often tedious and processing difficult due to the general low solubility of molecules having large aromatic networks. Second, as the energy difference between the ground and excited state decreases, the rate of internal conversion increases. This phenomena is often formulated as the energy gap law and is due to an increasing overlap of the wave functions between the zero vibrational level of the excited state and isoenergetic higher vibration levels of the ground electronic state.^5^

An opening for circumventing the energy gap law was recently presented using J-aggregates,^6^ which are ordered assemblies of dyes. The geometry of the assemblies is such that the transition dipole moments on individual dyes are coupled together by Coulomb interactions in a fashion that creates a common low-energy transition that is delocalized over the entire or parts of the aggregate.^7^ By delocalizing the excited state energy over several molecules, the reorganization energy, i.e., the energy difference of the excited state when nuclei are relaxed on the ground vs excited state surface, is considerably lower in the aggregate as compared to the parent molecule. This because each molecule only contains a fraction of the electron density of the excited state, resulting in smaller perturbations to the nuclear coordinates. Furthermore, apart from predicting an exponential increase in the rate of internal conversion with decreasing energy level difference, it is perhaps less known that the energy gap law also predicts a decrease of the vibrational relaxation rate with decreasing reorganization energy. Thus, systems that both reduce excited state energies and delocalize the energy, such as J-aggregates, offer an intrinsic physical compensation within the energy gap law that potentially could allow for highly emissive dyes in the NIR regime.

After the serendipitous discovery of J-aggregates in the 1930s,^8−10^ the interest in their photophysical properties has been emerging while finding practical applications, for instance in bioimaging,^11,12^ dye chemistry,^13^ and other areas of materials chemistry.^14−16^ The most well-known class of molecules forming J-aggregates is probably cyanine dyes,^17−20^ but examples covering many other dye classes exist, including BODIPY dyes,^21−23^ squaraine dyes,^24−28^ and porphyrins,^29−31^ among others.^32,33^ Rylenes is a class of dyes based on fused naphthalenes, and J-aggregate-forming examples include mainly perylene bisimides.^34−36^

Limited solubility is the major hurdle in the synthesis of longer oligorylenes and makes the investigation of their chemical and photophysical properties challenging. The low solubility of unsubstituted oligorylenes sets an upper size limit to terrylene for studies in solution. The naked quaterrylene core has been synthesized; however its low solubility prevents all types of solution-based analysis, for instance forcing the generation of the dication to perform NMR characterization.^37^ Several strategies have been proposed to increase its solubility; for instance cyclopenta ring-fused perylene^38^ as a building block allowed synthesizing curved rylene-based ribbons up to dodecarylene length.^39^ However, this strategy affects both intermolecular interactions that result in increased solubility and the electronic properties of the core. Introduction of bulky substituents such as *tert*-butyl^40^ and triflate moieties^41^ allowed obtaining soluble quaterrylenes, although with end-capping the ortho-positions, thus leaving no possibility for further elongation.

Here, we present a superradiant J-aggregate based on a bay-alkylated quaterrylene. The coupling between individual molecules within the aggregate is strong, causing the excited state to be shifted well into the near-infrared regime of the electromagnetic spectrum. Furthermore, the reorganization energy in the system is reduced due to delocalization, resulting in a slower rate of internal conversion in the aggregate as compared to the individual molecules. Thus, the electron delocalization in the supramolecular assembly counteracts the reduced energy gap within the energy gap law, with the result of a 4-fold increase in the emission quantum yield. Finally, we hypothesize that supramolecular assemblies in the form of J-aggregates offer a platform for highly emissive near-infrared systems and that bay-functionalization can be the method of choice for the synthesis of higher order linear oligorylenes, since it leaves the peri- and ortho- positions available for further derivatization.

## Results and Discussions

### Synthesis

**Scheme 1:**
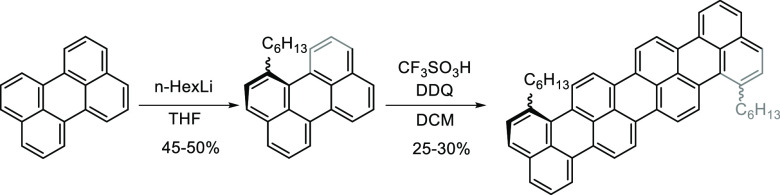
Synthesis of Dialkylated Quaterrylene; the Main Regioisomer, 1,1′-Dihexylquaterrylene, Is Shown

Bay-functionalization of perylene using alkyllithium introduces a twist to the symmetry plane of the long axis in perylene (Scheme 1).^42^ Consequentially, the broken symmetry plane lowers π–π stacking as shown by a higher solubility of bay as compared to ortho-alkylated perylene regioisomers, while retaining the intrinsic photophysical properties.^42,43^ This solubilization strategy can be applied to infinitely long oligorylene homologues because it leaves the ortho- and peri-positions available for further functionalizations. Two 1-hexylperylene units were coupled together using the Scholl reaction^44,45^ in order to obtain dihexylquaterrylene (Scheme 1; see Supplementary Section 2 for synthesis details). The reaction can occur on both short sides of perylene; hence the Scholl reaction can produce six different regioisomers. The high solubility of dihexylquaterrylene compared to quaterrylene allowed for NMR characterization (at 10^–5^ M in CD_2_Cl_2_), which concluded that the main product was the 1,1′-dihexylquaterrylene (see Supplementary Section 3 for structural elucidation).

**Figure 1:**
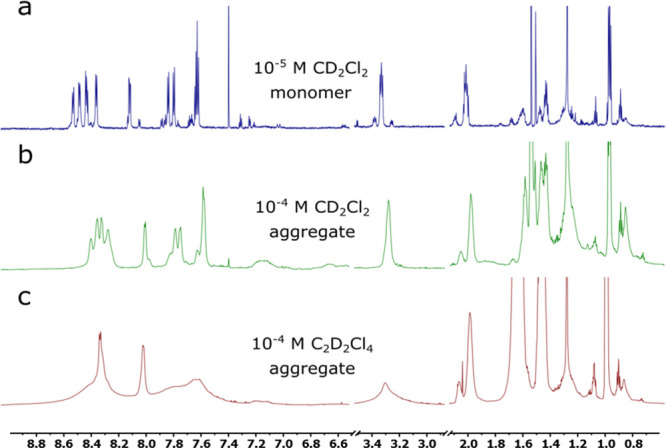
^1^H NMR spectra of dihexylquaterrylene: (a) 10^–5^ M dihexylquaterrylene in CD_2_Cl_2_ shows the monomeric species, (b) 10^–4^ M dihexylquaterrylene in CD_2_Cl_2_ shows the aggregate formation, (c) 10^–4^ M dihexylquaterrylene in C_2_D_2_Cl_4_ showing a higher tendency to aggregate as compared to (b).

While performing NMR characterization, an interesting observation was made. Monomeric dihexylquaterrylene could be observed at ∼10^–5^ M in CD_2_Cl_2_ (and in toluene-*d*_8_). However, signal broadening was observed at higher concentrations (∼10^–4^ M) in CD_2_Cl_2_, CDCl_3_, and C_2_D_2_Cl_4_ (Figure 1). While relatively similar features of aggregation were observed in CDCl_3_ and CD_2_Cl_2_, the spectrum recorded in C_2_D_2_Cl_4_ was different. The NMR spectrum of dihexylquaterrylene in C_2_D_2_Cl_4_ showed broader signals compared to CD_2_Cl_2_, which indicates the formation of larger/different structures in the C_2_D_2_Cl_4_ solution. Yet, the largest difference between the solvents was the visual appearance of the solutions. A complete discoloration of dihexylquaterrylene was observed when dissolved in C_2_H_2_Cl_4_. This large effect on the absorption spectrum can only occur by intermolecular interactions between the transition dipole moments in ordered aggregates, shifting the absorption to longer (J-aggregates) or shorter (H-aggregates) wavelengths than our eyes can detect.^7^

### Spectroscopy

**Figure 2:**
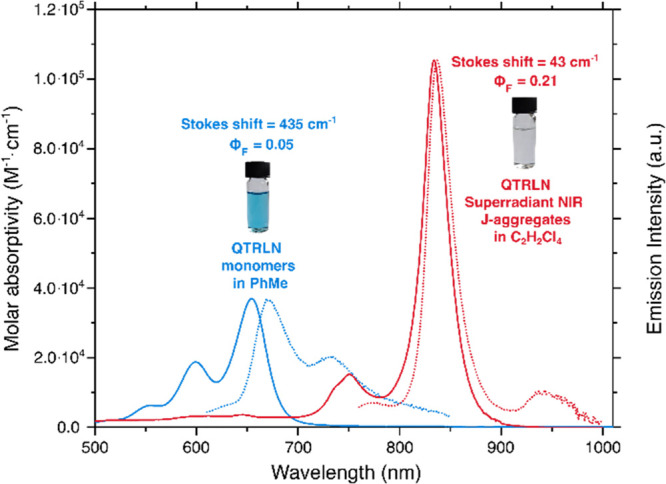
Absorption and emission spectra of 10^–6^ M dihexylquaterrylene recorded in toluene (blue) and in C_2_H_2_Cl_4_ (red) scaled according to their molar absorptivity values. Dihexylquaterrylene is present in its monomeric form in toluene, while in C_2_H_2_Cl_4_ it forms superradiant J-aggregates.

The improved solubility of dihexylquaterrylene compared to its unsubstituted analogue allowed for the visually observed discoloration phenomena to be spectroscopically studied in detail. The choice of solvent was used to promote either the monomeric (toluene or CH_2_Cl_2_) or aggregated (C_2_H_2_Cl_4_) species. As shown in Figure 2, the absorption spectrum of dihexylquaterrylene recorded in toluene exhibits rylene characteristic vibronic bands. The maxima of the vibrational progression A_0–0_, A_1–0_, and A_2–0_ of the S_1_ ← S_0_ transition are located at 654, 599, and 552 nm, respectively. Meanwhile, the maximum molar absorptivity in toluene is 37 000 M^–1^cm^–1^. As for the emission spectrum, it is nearly a mirror image of the absorption, with 0–0 and 0–1 peaks at 671 and 733 nm, respectively, demonstrating a Stokes shift of 15 nm (365 cm^–1^). The spectroscopic properties of dihexylquaterrylene in dichloromethane and chloroform are nearly identical to those in toluene. However, upon dissolving the dye in C_2_H_2_Cl_4_, discoloration occurs. This peculiar observation led us to further investigate the physical and photophysical changes that take place. The absorption of dihexylquaterrylene dissolved in C_2_H_2_Cl_4_ shows a large red-shift (180 nm or 3300 cm^–1^ vs dihexylquaterrylene dissolved in toluene) with a significantly narrowed absorption band. The number of vibrational bands observed decreases to two, and the ratio of the oscillator strengths of these two vibronic bands (A_0–0_/A_1–0_) increases. Furthermore, the molar absorptivity in C_2_H_2_Cl_4_ is nearly 3 times higher than that in toluene (105 000 vs 37 000 M^–1^ cm^–1^). The strong NIR emission observed has a mirror image relationship to the absorption. However, a mere 3 nm (43 cm^–1^) Stokes shift is observed. In conclusion, the combination of observed photophysical properties strongly suggests the formation of a J-aggregate when dihexylquaterrylene is dissolved in C_2_H_2_Cl_4_.

### Exciton Coupling Analysis

To gain quantitative structural insights on the nature of this J-aggregate, the values of the free-exciton bandwidth of the aggregate (*W*) and the nuclear relaxation energy of the monomer (*S*ω_vib_) were first compared. *W* is related to the degree of excitonic coupling between molecules in the aggregate and can be seen as a measure of delocalization.^46^ Meanwhile, *S*ω_vib_ represents the quantity of energy released when the molecule relaxes to the minimum of the excited state potential surface, which promotes disorder and thermal fluctuations resulting in localization.^47^ When *W* is greater than *S*ω_vib_, exciton delocalization outcompetes localization and the aggregate will enter the so-called strong coupling regime where the excited state of the individual molecules hybridizes to form one delocalized low-energy state.

*W* is proportional to the sum of resonant Columbic couplings between all molecular combinations in the aggregate. At the limit of nearest neighbor coupling in a linear aggregate, *W* can be calculated as^47^
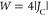
1where *J*_C_ is the Coulomb coupling between molecules in the aggregate. For a J-aggregate the Coulomb interaction is in phase, giving a lowest energy transition with enhanced transition probability. The coupling can be experimentally obtained from half the energy difference between the A_0–0_ of the monomer (dihexylquaterrylene dissolved in toluene) and the J-aggregate (dihexylquaterrylene dissolved in C_2_H_2_Cl_4_).^47^ This value is −1672 cm^–1^, and as a result, *W* is 6688 cm^–1^.

Meanwhile, *S*ω_vib_ represents the quantity of energy released when the molecule relaxes to the minimum of the excited state potential surface. Here, the Huang–Rhys factor (*S*) describes the vibrational coupling. It can be seen as a quantification of a relative shift between the ground and excited state potentials, and it was calculated by the ratio of the *I*_0–0_ and *I*_0–1_ intensities for monomer emission (the value is 0.5). Emission was chosen to reflect the coupling to the excited state surface, although a very similar value was received if the absorption spectra was used in the analysis. The vibrational energy, ω_vib_, is the energy separation between a promoting vibrational mode, and it was measured to be 1404 cm^–1^ as the difference between the spectrally well resolved A_0–1_ and A_0–0_ energies. Hence, *S*ω_vib_ is 700 cm^–1^. Given the values above, *W* is about 9.5 times larger than *S*ω_vib_, indicating that the J-aggregate is in the strong exciton coupling regime.^48^ As a consequence, the localized low-energy transition of dihexylquaterrylene is no longer present. Instead, the low-energy transitions of several dihexylquaterrylenes hybridize to form a single transition of much higher transition probability that is delocalized over many dihexylquaterrylenes.

We now turn our attention to the exciton delocalization length (*N*) of the J-aggregates. The exciton delocalization length, also known as the coherence number, describes the number of chromophores over which the exciton is coherently delocalized.^47^ It has been shown that the line width correlation between the monomer and J-aggregate follows a square root dependence on *N*.^49^ Furthermore, to minimize the influence of higher vibrational modes on the line width of the A_0–0_ or I_0–0_ transitions the full width at two-thirds maximum (FW^2^/_3_M) of the monomer and J-aggregate was used to determine *N*.^32^
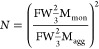
2

Using eq 2 and spectra in Figure 2, *N* is equal to 6 (both using absorption and emission), which should be interpreted as the low-energy transition of six dihexylquaterrylenes hybridizing to form one much stronger transition. Thus, the relatively large exciton delocalization length acquired through the line width is consistent with the system being in the strong exciton–exciton coupling regime acquired through the energy change of the electronic transition.

The increased transition dipole moment of emission in the J-aggregate (dihexylquaterrylene in C_2_H_2_Cl_4_) compared to the monomer (dihexylquaterrylene in toluene) also increases the radiative rate constant, an effect often referred to as superradiance. The emission quantum yields (Φ_F_) of dihexylquaterrylene were determined to be 0.21 and 0.05, in C_2_H_2_Cl_4_ and toluene, respectively. The latter goes in line with emission quantum yields previously observed for structurally rigid tetra-*tert*-butylquaterrylene derivatives,^32,41^ and the increase in Φ_F_ for the J-aggregate is therefore not likely to be due to aggregation-induced effects. Thus, Φ_F_ increased by a factor of 4 in C_2_H_2_Cl_4_, despite the large red-shift of the emission. Further, considering that the wavelength of emission is in the NIR regime, 0.21 is a very high Φ_F_ for a small organic molecule.^50,51^ For instance, J-aggregates of indocyanine green show Φ_F_ = 3 × 10^–4^ with an emission maximum at 890 nm,^52^ cyanine dyes have Φ_F_ = 0.05,^53^ and dibodipy J-aggregates demonstrate Φ_F_ = 0.03 at 920 nm.^54^

**Figure 3:**
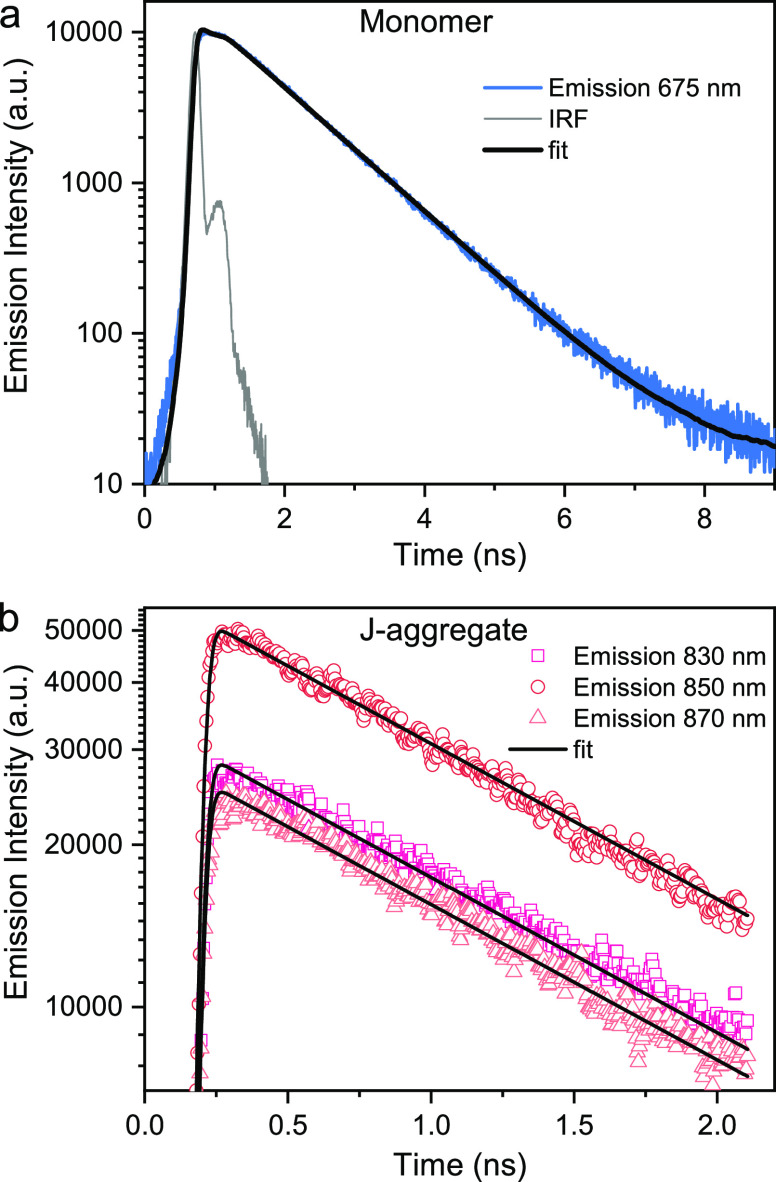
Time-resolved emission of monomer and J-aggregate: (a) Emission decay of the monomer together with the instrument response function (IRF). The black line shows the fit using a monoexponential decay model with pulse deconvolution, measured using time-correlated single photon counting, with an excitation wavelength of 660 nm. (b) Emission decay of the J-aggregate at various emission wavelengths. Black lines show global fits of the three time traces using a monoexponential decay model with pulse deconvolution, measured using streak camera detection, at an excitation wavelength of 840 nm.

Next, time-resolved emission spectroscopy was performed to obtain the excited state lifetime in order to analyze the radiative and nonradiative rates of the monomer and the J-aggregate excited states. The excited state lifetime of the monomer was determined to be 1.05 ns using the time-correlated single photon counting technique (Figure 3a). The emission decay for the J-aggregate was measured using a streak camera to be able to detect any wavelength dependencies in the decay that could result from inhomogeneities. Figure 3b shows time traces taken at three different emission wavelengths. The traces could be well represented by a globally fitted monoexponential function, indicating that the effect of any distribution of aggregate size and/or geometry is negligible as compared to signal-to-noise. Furthermore, no effect of the pump power on the decay could be observed (Figure S19), indicating that experiments were performed in a regime where the pump power was low enough that only one excited state was populated simultaneously on each aggregate. The emission quantum yields, and lifetimes were used to calculate the radiative and nonradiative decays. The results are shown in Table 1 and indicate that the higher Φ_F_ of the J-aggregate is a consequence from both a 3-fold increase in the radiative rate and a 40% decrease in the nonradiative one. The increased radiative rate, or superradiance, is a typical phenomenon in J-aggregates. For an ideal J-aggregate with monomers located in an ideal head-to-tail fashion, the radiative rate constants of the J-aggregate and the monomer, *k*_J_ and *k*_M_, respectively, relate with the delocalization length as^55^
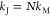
3

**Table 1:** Photophysical Properties of 1,1′-Dihexylquaterrylene^a^

	monomer toluene	J-aggregate C_2_H_2_Cl_4_
Abs_max_ [nm]	654	834
ε [M^–1^ cm^–1^]	37 000	105 000
Em_max_ [nm]	670	837
Stokes shift [cm^–1^]	365	43
τ [ns]	1.05	1.50
Φ_F_	0.05	0.21
*k*_r_ [s^–1^]	4.8 × 10^7^	1.4 × 10^8^
*k*_nr_ [s^–1^]	9.05 × 10^8^	5.33 × 10^8^

a The monomer was studied in toluene, and the J-aggregate was studied in C_2_H_2_Cl_4_.

Equation 3 suggest an *N* of 3. This value is lower as compared to that obtained by comparing the spectral shapes of emission (*N* = 6) and could be interpreted as molecules in the aggregate not being located in an ideal head-to-tail fashion. Instead, the molecules are most likely to be in a slip-stacked arrangement.

### Energy Gap Law

Previously, deviations from an exponential increase of the nonradiative rate in J-aggregates have been reported,^6^ but no decrease has to the best of our knowledge yet been seen. Typically, the vibrational relaxation accelerates as the emission red-shift, resulting in an increased rate of nonradiative relaxation. In other words, the reduced nonradiative relaxation for the J-aggregate indicates that the energy gap law was overcome in the system. To analyze the reason for the lower-than-expected nonradiative rate constant, we need to account both for the S_1_–S_0_ energy in the monomer and J-aggregate and for the effect that exciton delocalization causes on the nonradiative relaxation in the J-aggregate. Englman and Jortner, inspired by the golden rule treatment of nonradiative decay, derived the rate of relaxation from an electronically excited state to an isoenergetic high-energy vibrational mode of a lower electronic state (of any spin multiplicity, but we are only considering singlet states in the analysis).^56,57^ When the relative displacement between the two potential energy surfaces is small, the system is in the weak coupling limit (note, the coupling referred to here is between the ground and excited state). This is the regime for both the monomer and J-aggregate, as half the Stokes shift is considerable smaller than the energy of the promoting vibrational mode (365/2 vs 1404 cm^–1^ for the monomer). In this regime the potential energy surfaces do not cross, and the nonradiative decay of an organic dye can be described quantitatively as
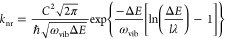
4where *C* is the effective electronic coupling constant between the excited state and ground state, Δ*E* is the energy difference between S_1_ and S_0_, ω_vib_ is the vibrational energy, *l* is the number of vibrational modes that induce the nonradiative transition, and λ is the reorganization energy of the promoting vibrational mode. Equation 4 describes the well-known energy gap law in which *k*_nr_ increases exponentially as the energy gap decreases. The value of λ can be estimated using the following equation for an arbitrary spectral shape,^58^
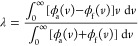
5where ν is the wavenumber and φ_a_ and φ_f_ are the area-normalized absorption and emission spectra, respectively. Using eq 5, λ_M_ of dihexylquaterrylene dissolved in toluene is 589 cm^–1^. Comparatively, the reorganization energy in the J-aggregate is much smaller, λ_J_ = 131 cm^–1^. The suppressed λ in the J-aggregate is a result of exciton delocalization, the reason being that each molecule in the aggregate only contains a fraction of the excited state electron density, resulting in a geometry-wise more ground state like excited state. The effect of delocalization on the reorganization energy can also be described using the monomer reorganization energy and the delocalization length:^6,59^
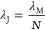
6

Accordingly, the exciton delocalization length was determined using eq 6 to be *N* = 4.5, which is in relatively good agreement with the values determined using the line width (*N* = 6) and superradiance (*N* = 3).

To quantitatively predict the nonradiative relaxation rate, the electronic coupling, *C*, must be known. This might be considerably different for the monomer and J-aggregate, preventing a quantitative assessment of *k*_NR_. Instead, we will semiquantitatively discuss the effect of Δ*E* and λ on *k*_NR_. Δ*E* occurs on three places in eq 4, two of which are inside the exponential function, with the result being a superexponential increase of *k*_NR_ with decreasing Δ*E*. λ on the other hand occurs only at one place inside the exponential function, and the decrease of *k*_NR_ with decreasing λ is therefore relatively smaller for λ compared to the increase with Δ*E*. However, the relative changes of Δ*E* and λ when going from the monomer to J-aggregate are 1.25 and 4.50, respectively (Table S3). The larger change in λ is therefore able to overcompensate for the reduced Δ*E* in eq 4, enabling the observed reduction in *k*_NR_ in the J-aggregate (Table 1). Thus, the semiquantitatively analysis above shows that exciton delocalization in the J-aggregate counterbalances the reduced energy gap and thereby substantially increases the emission quantum yield of this NIR-emitting dye. It is here interesting to do a thought experiment, that is, to assume that *C* is the same for the monomer and the J-aggregate and that *l* equals 1 for both, thus allowing for a quantitative analysis of the ratio of the nonradiative relaxation between the monomer and the J-aggregate. Using eq 4, this ratio equals 1600. For comparison, if the reorganization energy would have been the same for the J-aggregate as for the monomer, this ratio would have been only 0.0025, demonstrating how strongly λ affects the rate. The measured value of this ratio is 1.7, thus indicating that other factors that are nontrivial to experimentally acquire such as *C* and *l* also need to be taken into account in a quantitative analysis of the rate of internal conversion.

### Size of Aggregates

In a J-aggregate, the exciton does not need to be delocalized over the whole aggregate. The delocalization length therefore tells little about the actual size of the aggregate, although the physical size of the aggregate puts an upper limit on the theoretical delocalization length. In order to probe if the exciton delocalizes over the entire length of the physical aggregate, we turned to diffusion NMR. Diffusion NMR was conducted at the lowest possible concentration, i.e., at the detection limit for this experiment for the 900 MHz NMR system used. This translates to a molecular concentration of ∼10^–4^ M in C_2_D_2_Cl_4_, 2 orders of magnitude higher as compared to the UV/vis study. The rate of diffusion for the monomers (measured in CD_2_Cl_2_) is on the order of 10^–9^ m^2^/s, which is an expected order of magnitude for a small organic molecule in this solvent. In C_2_D_2_Cl_4_ on the other hand, the rate of diffusion for the aggregate is 2 orders of magnitude lower, thus confirming the aggregation-inducing ability of C_2_H_2_Cl_4_ (Table 2). It should also be noticed that indications of an exchange between monomers and aggregates could be observed by NMR (Figures S22 and S23, although no signs of monomers could be seen by UV/vis spectroscopy); aggregates formed are thus in dynamic equilibrium. Since the exchange is slow enough, it was possible to carry out the exchange suppression sequences (see Methods section in the Supporting Information) in order to determine diffusion coefficients for also the fast diffusion units (monomers). The diffusion of monomers was slower in C_2_D_2_Cl_4_ as compared to CD_2_Cl_2_, reflecting different viscosities of the solvents. The fast diffusion component did not show a large concentration dependence, which further supports the assignment of it to monomeric diffusion. The slower diffusion component did show a considerable concentration dependence; it was longer at higher concentrations, thus indicating a larger aggregate size at higher concentrations.

**Table 2:** Experimental Diffusion Coefficients of Quaterrylene

	monomer	J-aggregate
CD_2_Cl_2_ (10^–5^ M)	1 × 10^–9^ m^2^/s	
C_2_D_2_Cl_4_ (10^–4^ M)	2.6 × 10^–10^ m^2^/s	6 × 10^–11^ m^2^/s
C_2_D_2_Cl_4_ (>10^–4^ M)	2.2 × 10^–10^ m^2^/s	3.3 × 10^–11^ m^2^/s

To relate the rate of diffusion to the number of molecules in the aggregate, the geometry of the aggregate needs to be taken into account. The photophysical characterization concluded that a J-aggregate forms in C_2_H_2_Cl_4_; thus a slip-stacked arrangement of the molecules is expected. Typically, diffusion coefficients are translated to the hydrodynamic radius that describes diffusing units as ideal spheres. Such a model would provide large errors, considering the rod-like geometry of the quaterrylene in a slip-stacked arrangement. In an attempt to estimate the number of quaterrylene units in the aggregate, a model was built in which molecules were manually placed in a slip-stacked arrangement with an overlap of half a unit between the molecules and a π–π distance of 3.5 Å between the units.^43^ The created ellipsoid model of the aggregate was used to simulate a diffusion coefficient using the HYDRO++10 software (see Section 5 in the Supporting Information). The measured diffusion coefficients could in this manner be simulated using a model that included ∼14 and ∼34 quaterrylene units at concentrations of ∼10^–4^ and >10^–4^ M, respectively. This observation indicates a concentration dependence on the size of the aggregates that are formed in the solution. This is further supported by AFM and SEM micrographs (Figure S25) of drop-cast films showing, although quite homogeneous in size, much larger sized aggregates, indicating that aggregates continue to grow as the solvent evaporates. The size of aggregates was determined to be larger using diffusion NMR as compared to UV/vis. However, the NMR study was conducted at a higher concentration and the size of aggregates is expected to shrink when reducing the concentration. The extrapolated size range of aggregates using diffusion NMR therefore covers the size range as measured using UV/vis spectroscopy. Thus, the exciton is most likely delocalized over the whole aggregate at optical spectroscopy concentrations.

## Conclusion

Bay-derivatized quaterrylenes have drastically improved solubility as compared to the nonfunctionalized counterpart, which allowed us to study them in toluene solution. Moreover, aggregation was observed in C_2_H_2_Cl_4_, and photophysical investigations indicate the formation of J-aggregates. The quaterrylene J-aggregates show a 3-fold increase of the radiative rate constant, thus demonstrating superradiance. Further, a 40% decrease of the nonradiative rate constant was observed, which in combination with the increased radiative rate resulted in a 4-fold increase in the emission quantum yield, this despite a 180 nm red-shift of the absorbance maximum. This apparent violation of the energy gap law cannot be explained by aggregation preventing movements such as rotations in this rigid system. Instead, the key for explaining this unusual observation was found in the delocalized nature of the excited state of the J-aggregate. Delocalization reduces the reorganization energy, and the effect of the reorganization energy on the internal conversion has recently been highlighted.^6^ By considering the delocalized nature of the aggregate exciton the reduction of the nonradiative rate was explained using the framework of the energy gap law. Finding highly emissive dyes in the near-infrared regime has and will continue to be subjected to intense research because of their technological importance. However, finding such dyes is difficult because of the exponential increase of the nonradiative rate constant with decreasing energy of the excited state. We show here that delocalization can overcompensate for this exponential increase by reducing the reorganization energy, which can open up a new avenue for developing highly emissive dyes in the near-infrared regime of the electromagnetic spectrum.

## Supporting Information Available

Materials and methods, synthesis details, NMRs for spectral assignment and structure determination, streak camera image, and diffusion NMR and diffusion coefficient simulations (PDF)
