# Supplementary material for: Exciton Delocalization Counteracts the Energy Gap: A New Pathway toward NIR-Emissive Dyes
Source: J Am Chem Soc. 2021 Nov 8;143(45):19232–9. doi: 10.1021/jacs.1c10654 (PMC8603381; doi:10.1021/jacs.1c10654)
Supplement: Supplementary file 1 — ja1c10654_si_001.pdf [file ja1c10654_si_001.pdf]

Exciton delocalization counteracts the energy gap: A new pathway towards NIR-emissive dyes

Alexei Cravcenco,[1] Yi Yu,[1] Fredrik Edhborg,[2] Jonas F. Goebel,[1] Zoltan Takacs,[3] Yizhou Yang,[1] Bo Albinsson[2] and Karl Börjesson[1*]

[1]Department of Chemistry and Molecular Biology, University of Gothenburg, Kemivägen 10, 41296 Gothenburg, Sweden
[2]Department of Chemistry and Chemical Engineering, Chalmers University of Technology, Kemivägen 10, 41296 Gothenburg, Sweden
[3]Swedish NMR Centre, University of Gothenburg, Medicinaregatan 5C, 40530 Gothenburg, Sweden

Contents:

Contents: 1
1. Methods 2
General Synthesis 2
General optical spectroscopy 2
General AFM and SEM 3
General diffusion NMR and diffusion coefficient simulations 3
2. Synthesis 5
3. Spectral assignment and structure determination 7
4. Photophysical data 18
5. Diffusion NMR and diffusion coefficient simulation 19
6. AFM and SEM images 24
7. References 25

1. Methods

General Synthesis

All starting materials were purchased from Sigma-Aldrich Chemical Co. and used without further purification unless otherwise noticed. All moisture- and oxygen-sensitive reactions were carried out using Schlenk techniques in oven-dried glassware. Solvents used for moisture and oxygen-sensitive reactions were dried using a PS-MD-5/7 Inert technology purification system and, if necessary, degassed by freeze-pump-thaw cycles and stored over 4 Å molecular sieves under argon atmosphere. ^{1}H NMR (nuclear magnetic resonance) spectra were recorded on Bruker spectrometers at 900 MHz, 800 MHz or 600 MHz, ^{13}C NMR spectra were recorded at 201 MHz using $CDCl_3$, CD_2Cl_2 or 1,1,2,2-tetrachloroethane-d_4 ($C_2D_2Cl_4$). All three magnets were equipped with a Bruker AVENCE III-HD console. The 900 MHz magnet had a 3 mm TCI H-C/N-D cryoprobe inserted while the 800 was equipped with a 5 mm TXO C/N-H-D, ^{13}C optimized cryoprobe. The 600 MHz magnet had a room temperature BBI probe inserted. The maximum current of the Z-gradient was 10 A in all the systems. J-coupling values are given in Hertz, and chemical shifts are given in parts per million using tetramethylsilane, with 0.00 ppm as an internal standard. High-resolution MS was obtained from an Agilent 1290 infinity LC system equipped with an auto sampler in tandem with an Agilent 6520 Accurate Mass Q-TOF LC/MS.

General optical spectroscopy

The absorbance spectra were measured using a Perkin Elmer LAMBDA 950 spectrophotometer. Molar absorptivities were calculated as an average of three individual measurements. About 1 mg was weighed out in each measurement and then dissolved in either toluene (monomer) or TCE (J-aggregate). Emission measurements were performed using an FLS1000 (Edinburgh Instruments) spectrofluorometer. Emission quantum yields were measured using the relative method using quinine sulfate (Sigma-Aldrich) in a 0.1-N solution of H_2SO_4 (quantum yield of quinine sulfate, 0.59[1]) as an internal standard for perylenes, Oxazine 1 (Sigma-Aldrich) in ethanol solution (quantum yield of Oxazine 1, 0.15[1]) as internal standard for dihexylquaterrylene monomers and IR-140 (Sigma-Aldrich) in ethanol solution (quantum yield of IR-140, 0.167[2]) as internal standard for dihexylquaterrylene J-aggregates. Emission quantum yields are reported as an average of three samples. The fluorescence lifetime of the monomer was determined using time-correlated single-photon counting using an Edinburgh Instruments LifeSpec II equipped with an MCP-PMT detector and a 660 nm pulsed diode laser (PicoQuant) as excitation source. The fluorescence lifetime of the J-aggregate was determined using a streak camera (C5680, Hamamatsu) equipped with a spectrometer (Acton SP2300, Princeton Instruments) and a synchroscan sweep unit (M5675, Hamamatsu). The excitation source was a picosecond pulsed, 80 MHz, Ti:sapphire laser (Tsunami, Spectra-Physics) pumped by a Millennia Pro X laser (Spectra-Physics). Emission decay traces at various wavelengths were obtained from the streak

camera images by integrating over narrow wavelength ranges of 5-10 nm. The decay traces were fitted to single exponential decays using instrument response deconvolution. The instrument response function (IRF) was obtained by fitting the time profile of the scattered light from the laser pulse to a gaussian function, yielding an IRF with an FWHM of approximately 40 ps. All sample concentrations, for both emission and lifetime measurements, were set to a maximum of 0.1 absorbance unit to avoid inner filter effects.

General AFM and SEM

The diameter and size of aggregates was measured by atomic force microscopy (AFM). A solution of the aggregates was drop casted on a silicon wafer. After evaporation of the solvent, the AFM scan was conducted on a NT-MDT NTEGRA AFM in tapping mode using a silicon cantilever (Tap150Al-G from Budget Sensors). By the same sample preparation, the aggregates were observed under scanning electron microscopy (SEM) using a Zeiss Ultra 55 scanning electron microscope with an acceleration voltage of 15 kV.

General diffusion NMR and diffusion coefficient simulations

The diffusion coefficients were measured with the *ledbpgp2s*[3] (available in default Bruker library) and with the PROJECTED[4] exchange suppressed pulse program. Due to the shorter T_2 relaxation time of the aggregate the PROJECTED sequence can only be employed to measure the methyl group in dihexylquaterrylene. The gradient was set to the smoothed square shape (SMSQ10.100), which is included in the Bruker gradient shape library. The pulse program was extended to include multiple solvent suppression. The gradient calibration constant (GCC) was determined using a doped water sample. The big delta (d20) was set to 50 ms in the experiments recorded with the *ledbpgp2s* pulse sequence expect for the high concentration $C_2D_2Cl_4$ sample where it was set to 60 ms and to 20 ms (d2=40 ms) in the PROJECTED sequence with n=8. The little delta had to be set differently for 1-hexylperylene, dihexylquaterrylene and the aggregate. The little delta for the dihexylquaterrylene in the PROJECTED sequence was set to 1.8 ms.

The diffusion data were fitted by a python script using the MINUIT - MINGRAD routine from the iminuit package. The error was assessed in two main ways. Firstly, the standard deviation of the noise of each slice of the pseudo 2D measurement was determined. This served as the error for each point in the integral region. The integral was formed as the sum of intensities within a defined region. The error of the integral was then determined by the square root of the sum of the squared errors. The quality of fit was assessed using the Variance Reduction method.[5] Secondly, the error in the fitted diffusion coefficients were assessed. If multiple peaks were available for the measurement of the same compound then the average was taken and the confidence interval was calculated from the separate measurement rather than fitting them all in the same time. This is only possible if the same diffusion coefficient can be determined using multiple sites in the molecule. This was not fulfilled for the aggregate where only

one region can be used. In this case the Monte Carlo error estimation was employed. The fit was repeated 10000 times to "synthetic" data sets. These data sets were generated from the original using the error of the point determined as described above by randomly generating a point with normal distribution around the particular measured integral. The diffusion coefficient then is the average of the 10000 runs.

Diffusion coefficients were simulated using HYDRO++10,[6, 7] HYDROSUB[8] and HYDROPRO.[9] The simulation results were nearly identical for all three softwares, thus only one example (HYDRO++10) is given. Solvent viscosity was specified as 0.00413 Poise[10] for CD_2Cl_2 and 0.0165 Poise[11] for $C_2D_2Cl_4$ at 25 °C when input files were generated.

2. Synthesis

a) **1-hexylperylene** was synthesized according to the literature procedure developed earlier by our group.[12] NMR shifts and other spectral data was in line with those reported earlier. In addition to the previous study, a full spectral assignment has been carried out for 1-hexylperylene *vide infra.*

b) **1,1'-dihexylquaterrylene**

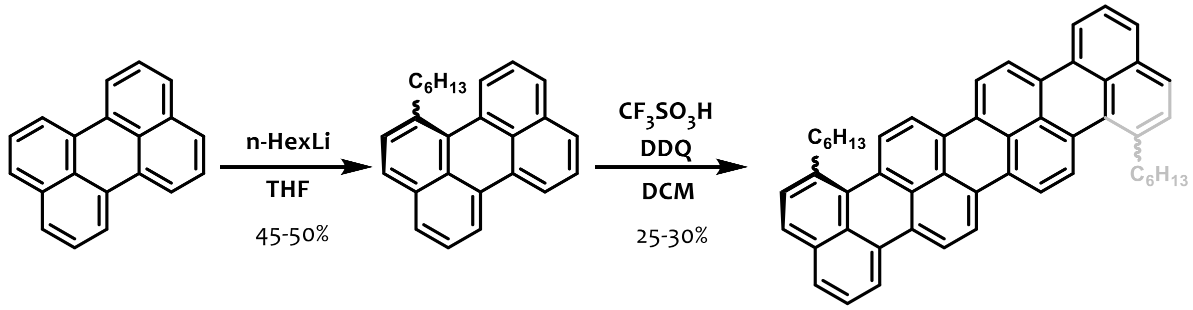

Figure S1. The synthesis of 1,1'-dihexylquaterrylene.

In a microwave vial 1-hexylperylene (30.8 mg, 0.1 mmol) was placed and dissolved in CH_2Cl_2 (4 mL). After 10 min, DDQ (22.7 mg, 0.1 mmol) and triflic acid (9 µL, 0.1 mmol) were added and the microwave vial was sealed with a cap and sparged with nitrogen for 20 min. The reaction mixture was then stirred overnight at room temperature. The resulting mixture was quenched with diisopropylethylamine (1 mL) and passed through a column containing basic aluminium oxide eluting with (20/80 ratio of toluene/cyclohexene). The collected green fractions were concentrated and purified by a size exclusion chromatography using BioBeads SX-3 as a stationary phase and CH_2Cl_2 as an eluent. The collected blue fractions were concentrated under reduced pressure to afford the title compound as a dark purple solid 9.3 mg, 28% yield (85:15 isomeric ratio). The chemical structure of the minor isomer was not assigned due to insignificant amounts.

^{1}H NMR (CD_2Cl_2, 900 MHz): δ = 8.44 (d, *J* = 8.3 Hz, 1H, **m**), 8.39 (d, *J* = 7.9 Hz, 1H, **n**), 8.34 (d, *J* = 8.2 Hz, 1H, **l**), 8.27 (d, *J* = 7.3 Hz, 1H, **k**), 8.03 (d, *J* = 7.9 Hz, 1H, **o**), 7.75 (t, *J* = 7.9 Hz, 7.3Hz, 1H, **i**), 7. 71 (d, *J* = 8.3 Hz, 1H, **h**), 7.55 (t, *J* = 7.8 Hz, 7.3 Hz, 1H, **j /overlap g**), 3.26 (m, 2H, **f**), 1.96 (m, 2H, **e**), 1.58 (m, 2H, **d**), 1.46 (q, *J* = 6.6 Hz, 2H, **c**), 1.41 (s, *J* = 7.4 Hz, 2H, **b**), 0.96 (t, *J* = 7.4 Hz, 3H, **a**) ppm.

^{13}C NMR (CD_2Cl_2, 225 MHz): δ = 131.39, 128.1, 127.74, 127.46, 126.32, 121.71, 121.62, 121.28, 121.05, 36.70, 32.2, 31.54, 30.00, 23.17, 14.26 ppm.

HRMS (APCI+): *m/z* calcd. for $C_{52}H_{44}$: 669.3516, found: 669.3499

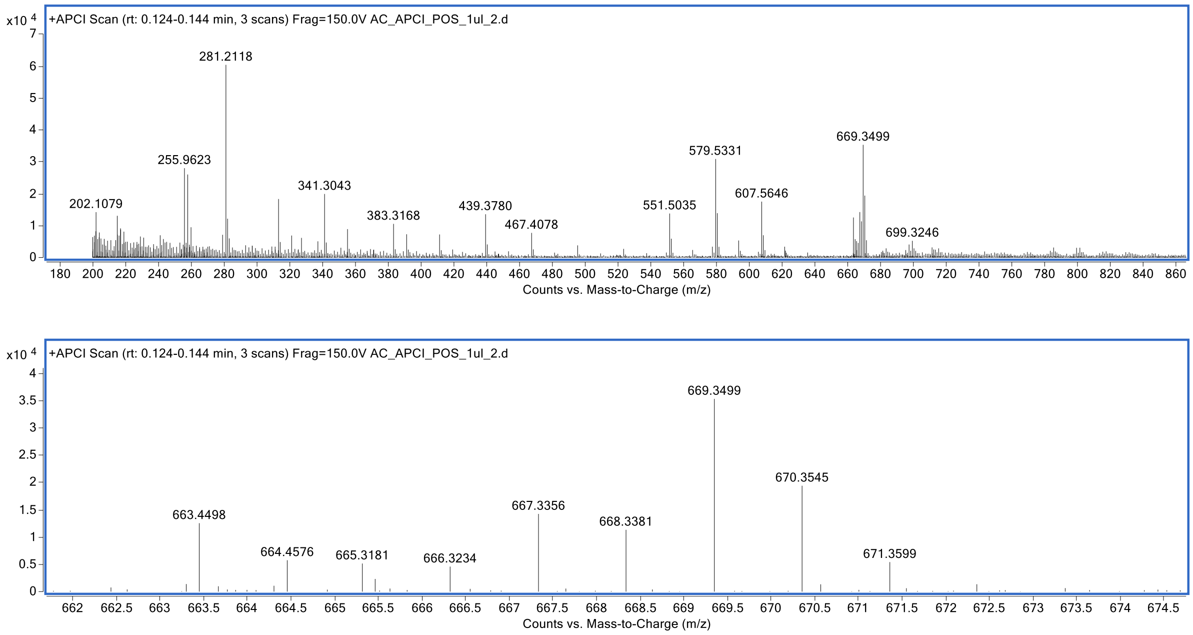

Figure S2. HRMS (APCI+) spectra of 1,1'-dihexylquaterrylene.

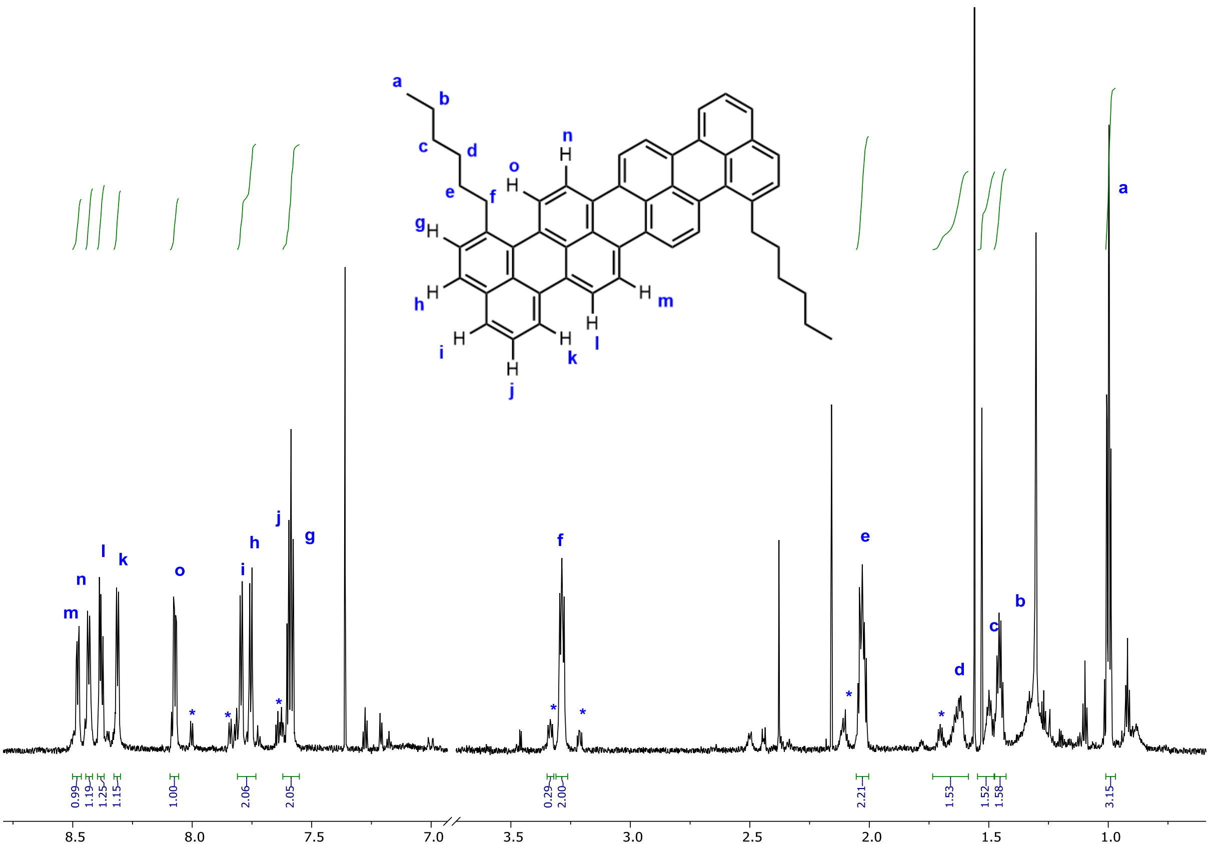

Figure S3. ^{1}H NMR spectrum of 1,1'-dihexylquaterrylene recorded at 900 MHz, region between 3.7-6.9 ppm disregarded due to lack of relevant signals. Minor regioisomer (ca. 15%) signals marked with asterisk.

3. Spectral assignment and structure determination

a) 1-hexylperylene

Spectral assignment was carried our for 1-hexylperylene and it was based on HSQC (Fig S5), 2D anti-Z-COSY[13] (Fig. S6), TOCSY, and IMPACT-HMBC[14] spectra. The protons are labelled with letters and quaternary carbons are labelled with digits in the figures. The experiments were carried out at 25 °C in CD_2Cl_2. The chemical shifts and proton/carbon spectral assignment for 1-hexylperylene are presented in Table S1. This information was used as an aid when making spectral assignment for 1,1'-dihexylquaterrylene *vide infra*.

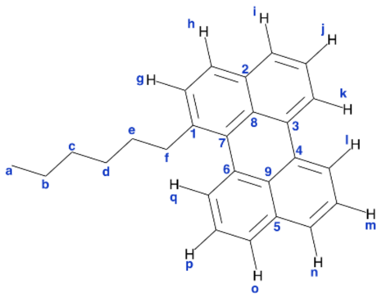

Figure S4. The nuclei labelling in 1-hexylperylene.

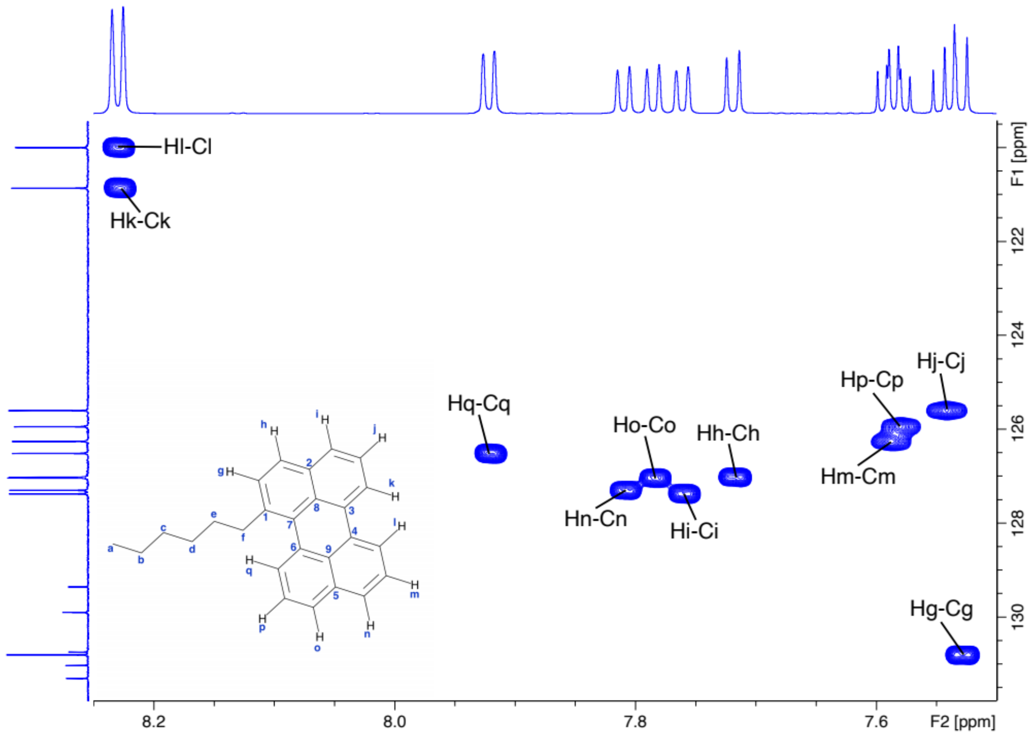

Figure S5. The aromatic part in the HSQC spectrum of 1-hexylperylene.

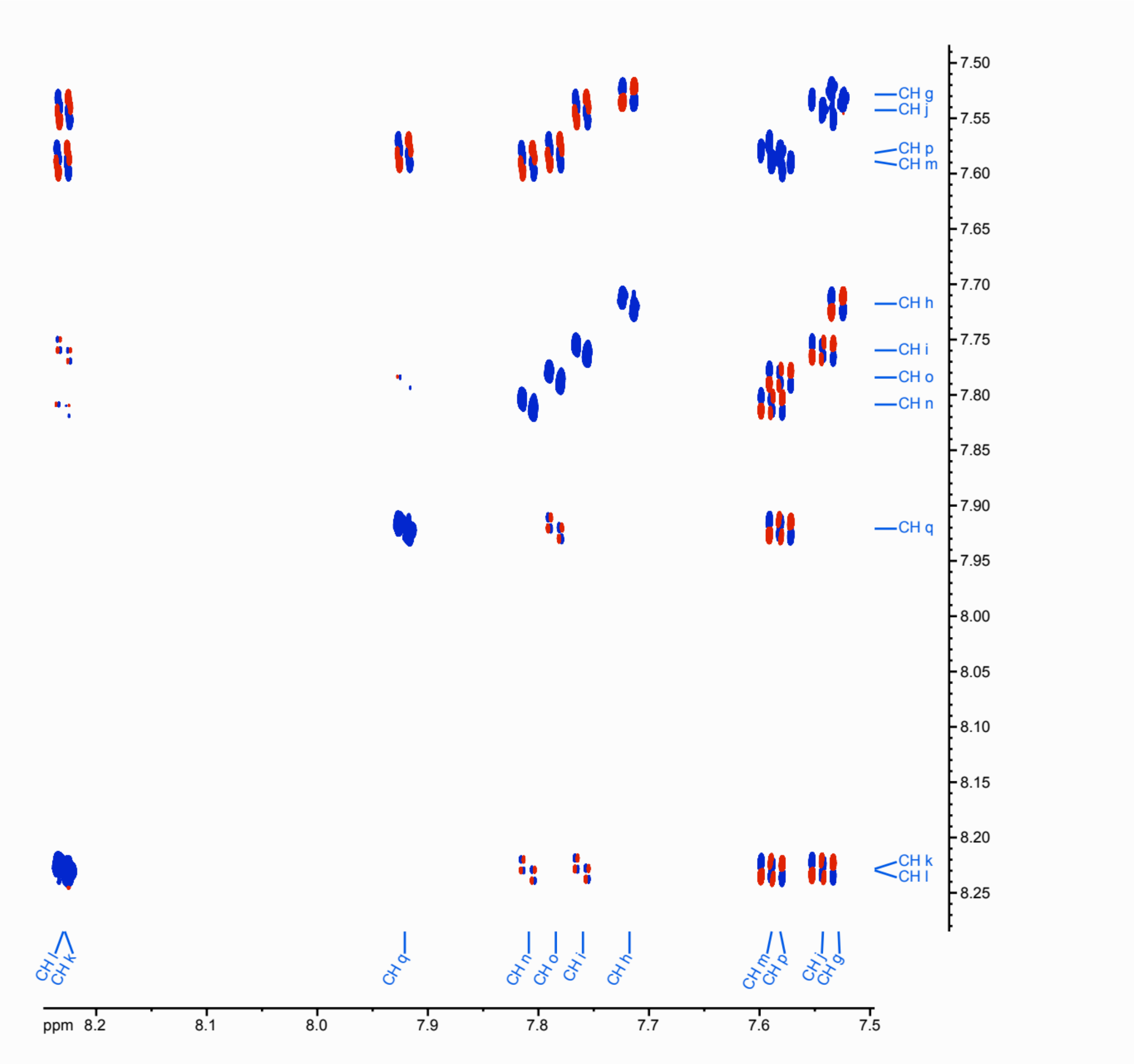

Figure S6. The spectral assignment for 1-hexylperylene in the aromatic part of an Anti-Z-COSY recorded at 800MHz in CD_2Cl_2.

Table S1. The spectral assignment of 1-hexylperylene at 25 °C in CD2Cl2. The spectra have been recorded at 800MHz.

Nucleus	Chemical shift (ppm)<br>$^{1}H/^{13}C$	Note<br>appearance of ^{1}H spectra
Ha/Ca	0.97 / 13.87	Triplet
Hb/Cb	1.42 / 22.68	Multiplet
Hc/Cc	1.45 / 31.69	Multiplet
Hd/Cd	1.56 / 29.53	Multiplet
He/Ce	1.97 / 30.97	Multiplet
Hf/Cf	3.20 / 36.04	Multiplet
Hg/Cg	7.53 / 130.79	Doublet
Hh/Ch	7.72 / 127.03	Doublet
Hi/Ci	7.76 / 127.36	Doublet
Hj/Cj	7.54 / 125.59	Triplet
Hk/Ck	8.23 / 120.85	Doublet (overlap with l)
Hl/Cl	8.23 / 119.99	Doublet (overlap with k)
Hm/Cm	7.59 / 126.25	Triplet
Hn/Cn	7.81 / 127.29	Doublet
Ho/Co	7.78 / 127.01	Doublet
Hp/Cp	7.58 / 125.93	Triplet
Hq/Cq	7.92 / 126.50	Doublet
1	NaN / 138.76	Quaternary C
2	NaN / 132.67	Quaternary C
3	NaN / 130.73	Quaternary C
4	NaN / 131.02	Quaternary C
5	NaN / 134.21	Quaternary C
6	NaN / 131.29	Quaternary C
7	NaN / 129.35	Quaternary C
8	NaN / 129.89	Quaternary C
9	NaN / 129.89	Quaternary C

b) 1,1’-dihexylquaterrylene

In order to determine the main reaction product of the Scholl condensation, a detailed NMR evaluation of the reaction product was performed at 10^{-5} M in CD_2Cl_2. As an aid in the analysis are the chemical structure of all plausible regiosisomers presented in Figure S7. The assignment was based on 2D TOCSY, 2D/1D NOESY and HSQC spectra. HMBC spectra could not be recorderd within a reasonable time given the very low concentration of the sample. For the 1D proton, 2D TOCSY and NOESY, the solvent and water peaks had to be suppressed in order to maximize the receiver gain. This was achieved by shaped pulse presaturation. The shaped pulse was set up by WAVEMAKER.[15]

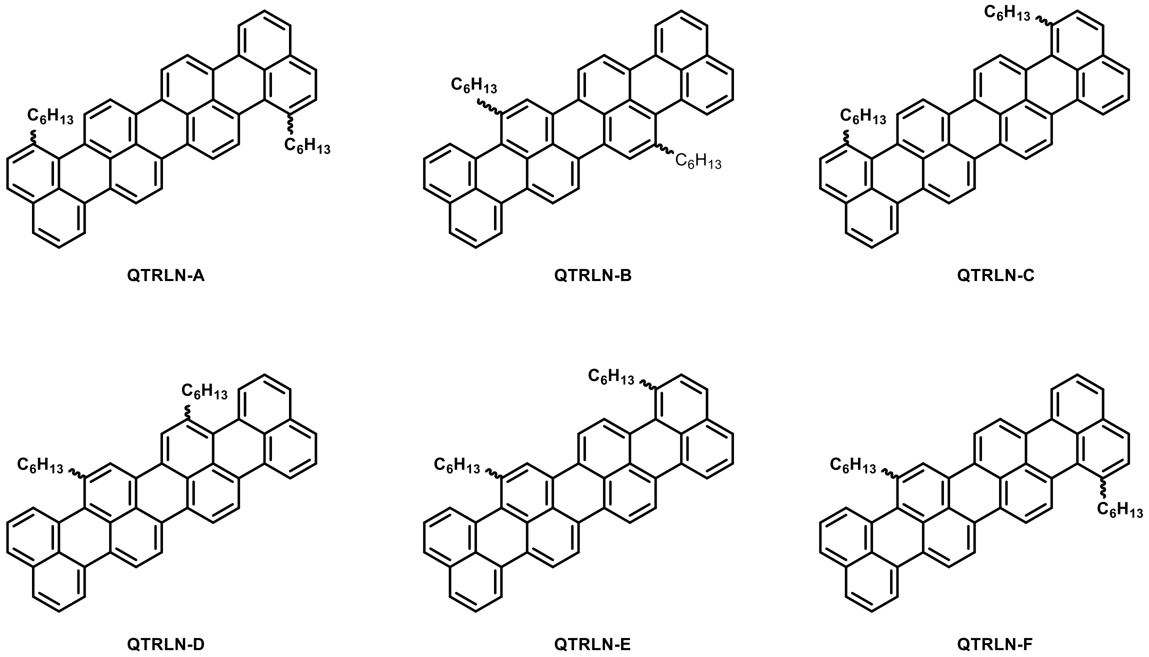

Figure S7. The chemical structures of all the plausible dihexylquaterrylene regioisomers that could be produced during the Schöll condensation of 1-hexylperylene.

From the proton spectrum it is evident that the reaction product is dominated by a single regioisomer and also contains minor isomers (Fig. S9-10). Through a series of NMR experiments the structure of the main regioisomer was derived as 1,1’-dihexylquaterrylene (QTRLN-A or QTRLN-C as they are magnetically equivalent and undistinguishable with NMR). Hereafter, 1,1’-dihexylquaterrylene will be referred to as the main product and QTRLN-A will be shown in schemes. The proton labelling is shown in Figure S8. The details of the spectral assignment are outlined below.

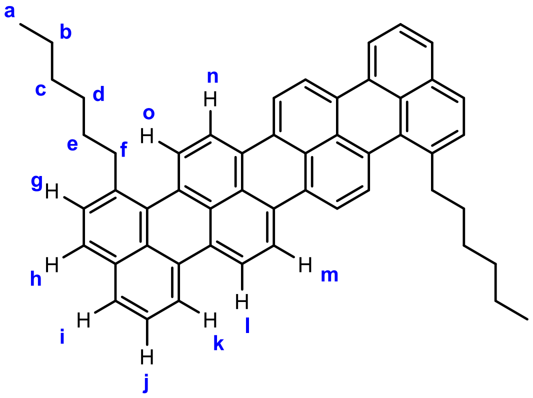

Figure S8. The proton labelling in 1,1'-dihexylquaterrylene.

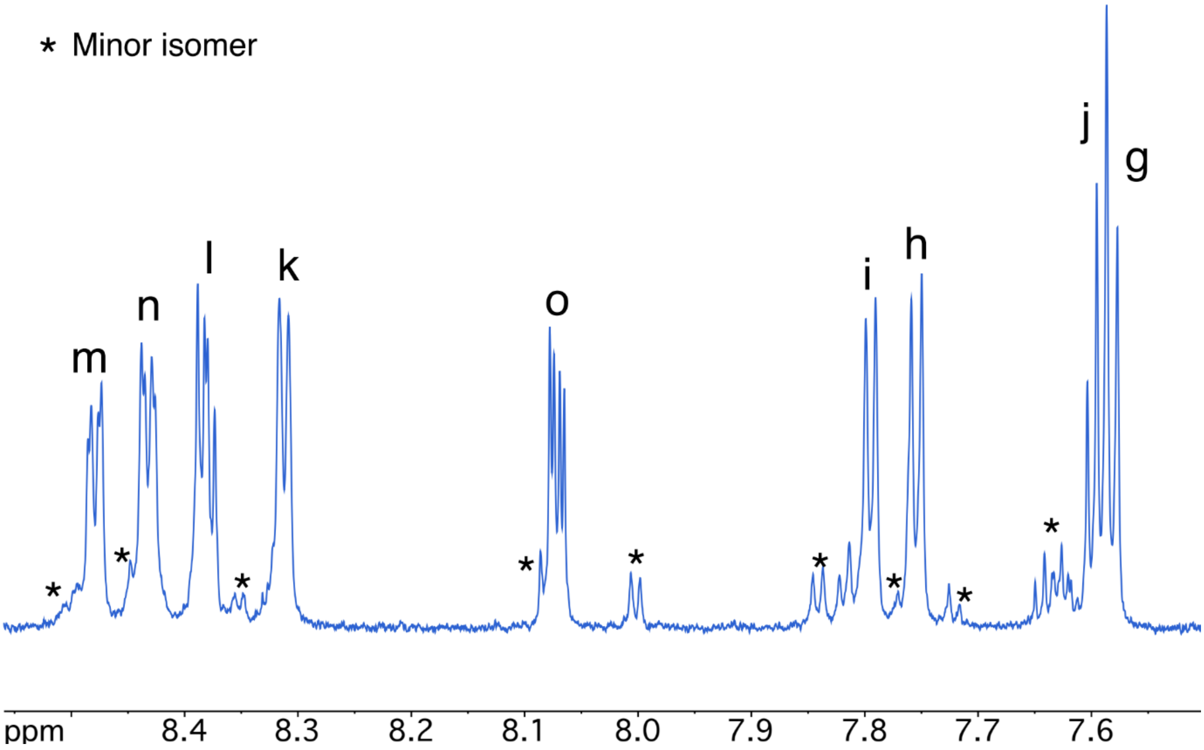

Figure S9. ^{1}H NMR spectrum showing aromatic region of the 10^{-5} M dihexylquaterrylene recorded at 900 MHz in CD_2Cl_2 at 25 °C. Minor regioisomer signals are marked with asterisk. The $CHDCl_2$ and water peaks were suppressed by shaped pulse presaturation.

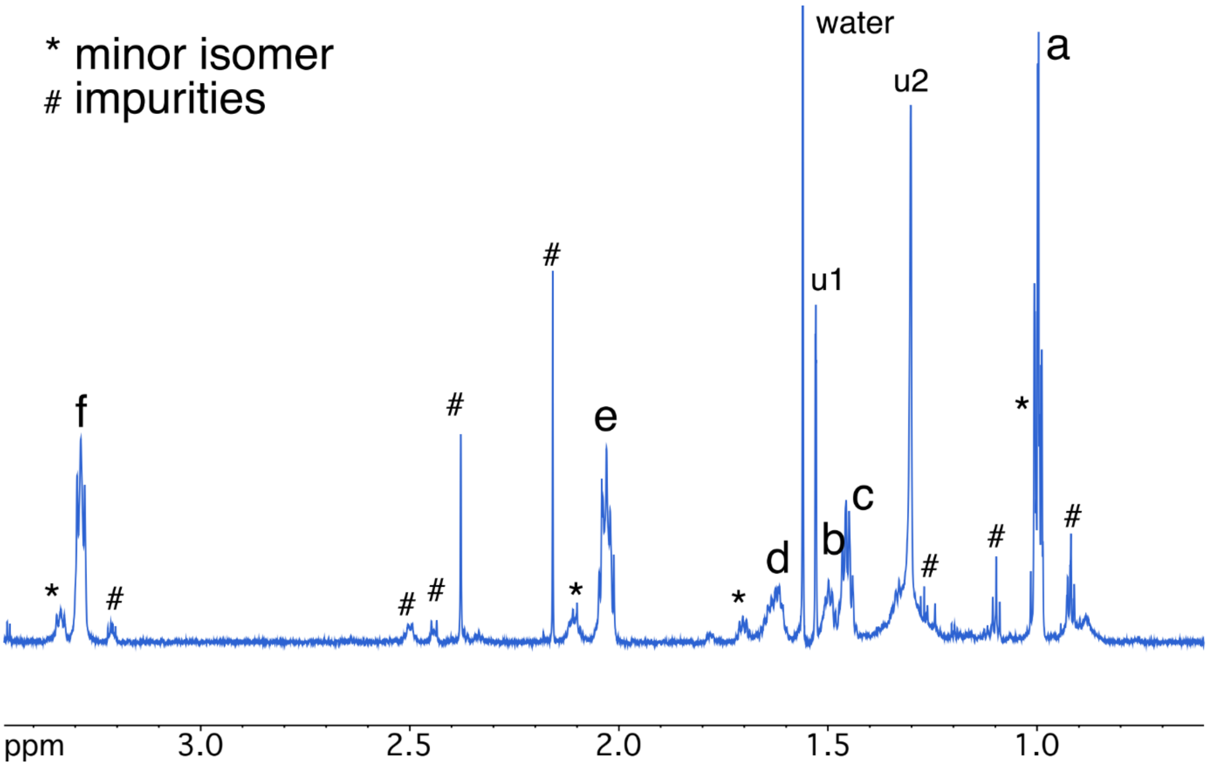

Figure S10. ^{1}H NMR spectrum showing the aliphatic region of the 10^{-5} M dihexylquaterrylene recorded at 900 MHz in CD_2Cl_2 at 25 °C. Minor regioisomer signals are marked with an asterisk (*), and solvent impurities are marked with a hashtag (#). Signals that were not identified are marked as u1 and u2 (CH_2). The $CHDCl_2$ and water peaks were suppressed by shaped pulse presaturation.

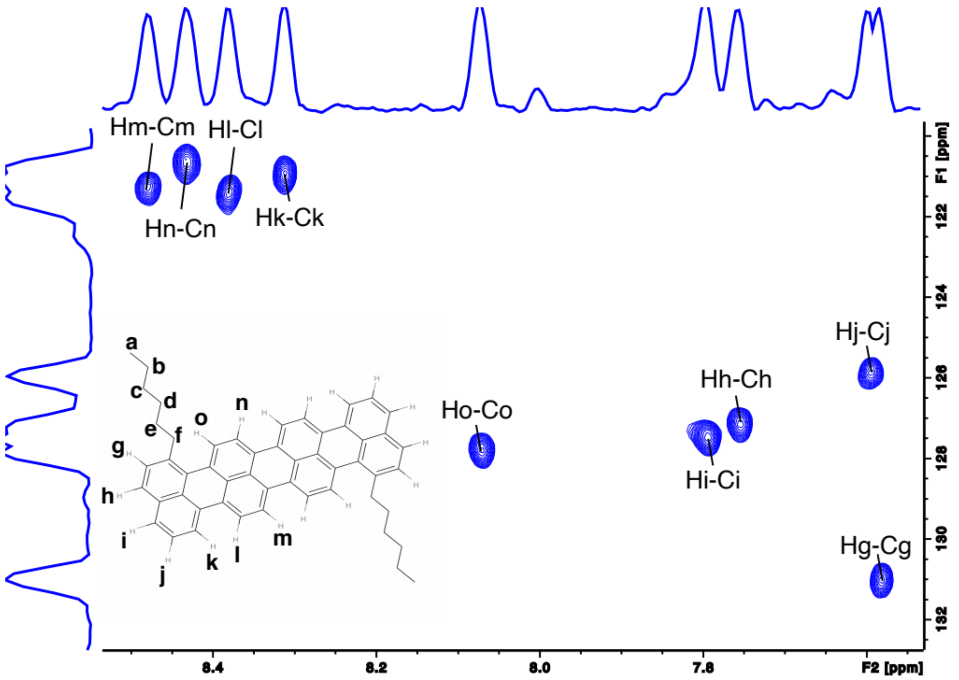

Figure S11. The aromatic part of the HSQC spectrum of dihexylquaterrylene. There are 9 peaks in the spectrum confirming that all the carbons attached to aromatic protons are accounted for.

The HSQC spectrum shown in Figure S11 describes correlations between proton signals and corresponding carbons in dihexylquaterrylene. The aromatic part is represented by 9 protons that are coupled to the carbons and from this spectrum it is evident that all the signals are accounted for. Several signals were assigned with an aid of the HSQC spectrum of 1-hexylperylene (Fig. S5). Peaks **u1** and **u2** (Fig. S10) could not be identified but **u2** is a CH_2 group according to the edited HSQC with no coupling partners or it only has very small couplings to other protons. It is interesting to note that **u2** diffuses with a very similar diffusion coefficient to the dihexylquaterrelyene monomer if not identical (Fig S20-21). At this low concentration, no HSQC peak could be seen for the peak **u1** that appears as a proton coupled to a deuterium or another spin 1 nucleus.

The 2D-TOCSY spectrum describe correlations within spin systems and is shown in Figure S12. Green solid squares represent two spin systems, while dashed black squares represent 3 spin systems.

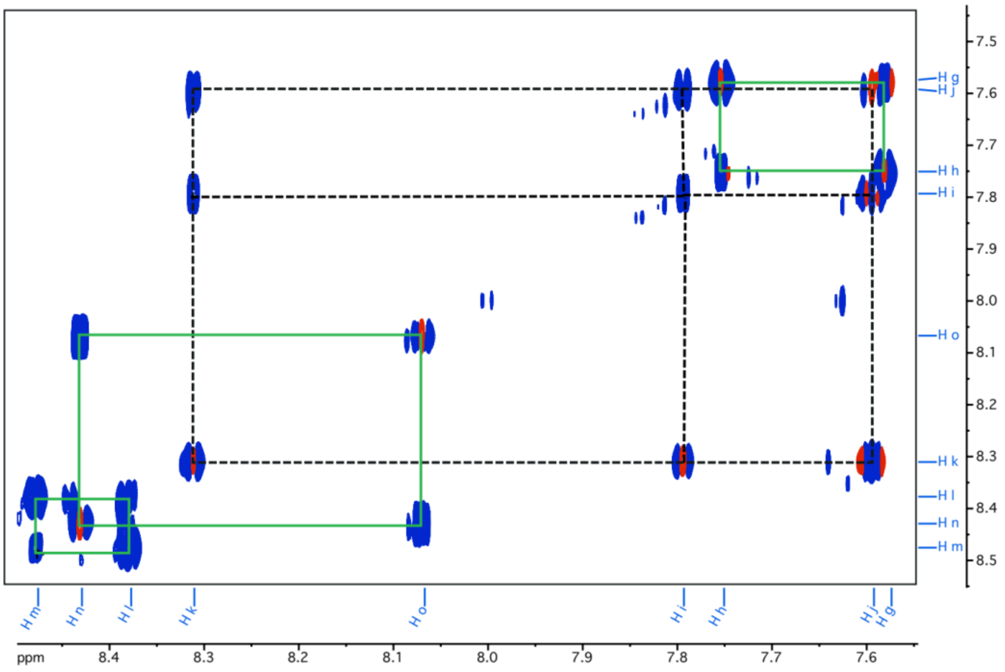

Figure S12. Aromatic region of the 2D-TOCSY spectrum showing the correlations between spins within a spin system in the major isomer. Green squares represent 2 spin systems and black dashed squares represent the 3 spin system.

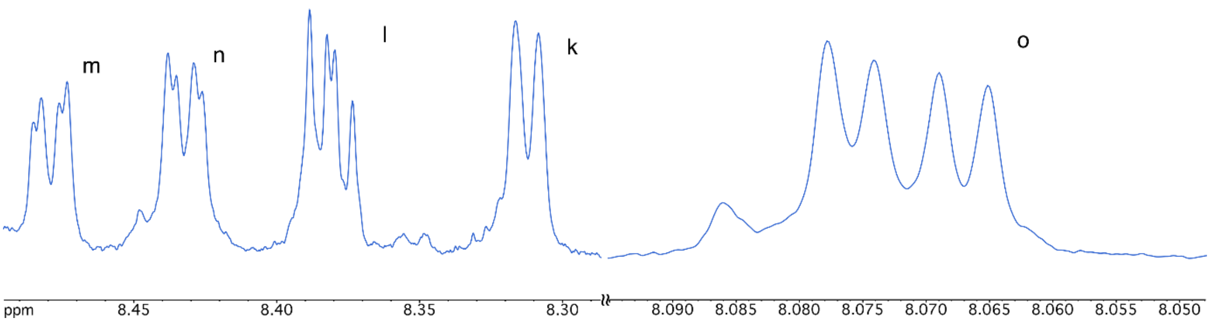

Figure S13. Part of the ^{1}H spectrum showing the protons o,k,l,n,m in the aromatic region in the 10^{-5} M dihexylquaterrylene. The spectrum was recorded at 900 MHz in CD_2Cl_2 at 25 °C. The $CHDCl_2$ and water peak were suppressed by shaped pulse presaturation.

Figure S13 shows the aromatic protons **o**, **k**, **l**, **m** and **n**. It can be seen that **o**, **l**, **n** and **m** seemingly display extra coupling because they appear as doublet of doublets. This however cannot be seen in the TOCSY spectrum (Fig. S12), meaning no more than one coupling partners could be identified for **o**, **l**, **n** and **m**. It is thus highly probable that the two perylene rings within the quaterrylene scaffold are not completely equivalent. It is also interesting to note that the CH_3 protons appear as a “doublet of triplets” (Fig. S14) which supports the hypothesis of perylene units within quaterrelene being magnetically non-equivalent. This is not surprising, given the possibility of atropisomeric exchange that was seen in case of 1-hexylperylene[11].

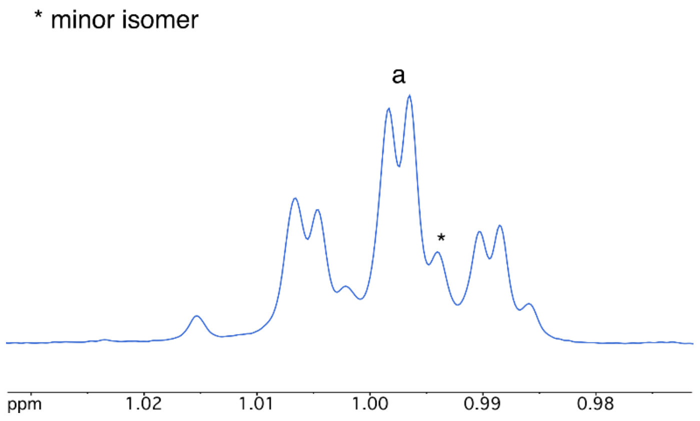

Figure S14. The CH_3 of dihexylquaterrylene at 900MHz in CD_2Cl_2 at 25 °C. The "doublet of triplet" structure is clearly visible. Lower intensity triplet behind the main peak belongs to the minor regioisomer.

It is interesting to note that the doubling of the peaks belonging to proton **o** disappears in $CDCl_3$ (Figure S15). Other peaks also show this effect, and this suggests that it is related to dynamics of the ring system. This is shown in Figure S15 where it is seen that possible exchange is affected by the solvent. The coupling constant between proton **o** and **n** is 8 Hz in both solvents.

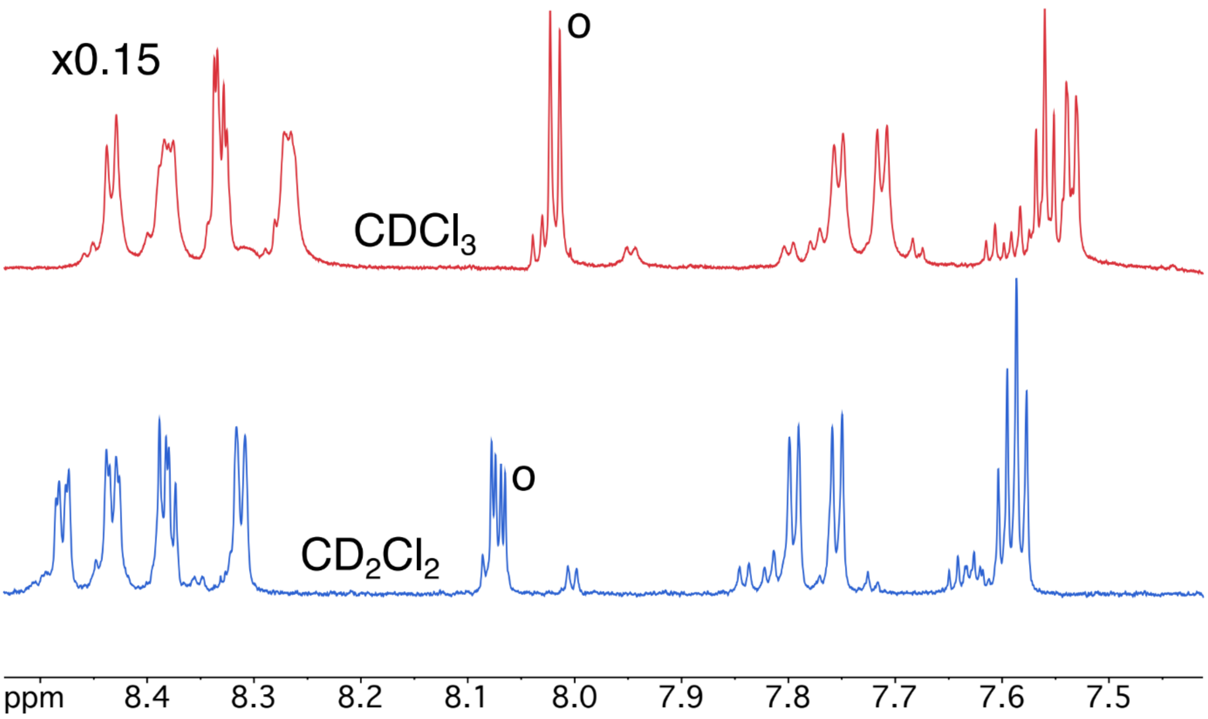

Figure S15. The comparison of the aromatic part of the ^1H spectrum of 10^{-5} M dihexylquaterrylene recorded at 900 MHz at 25 °C. The blue spectrum is in CD_2Cl_2 and was recorded with 128 scans and the red spectrum is $CDCl_3$ and was recorded with 4096 scans. The solvent and water peaks were suppressed by shaped pulse presaturation.

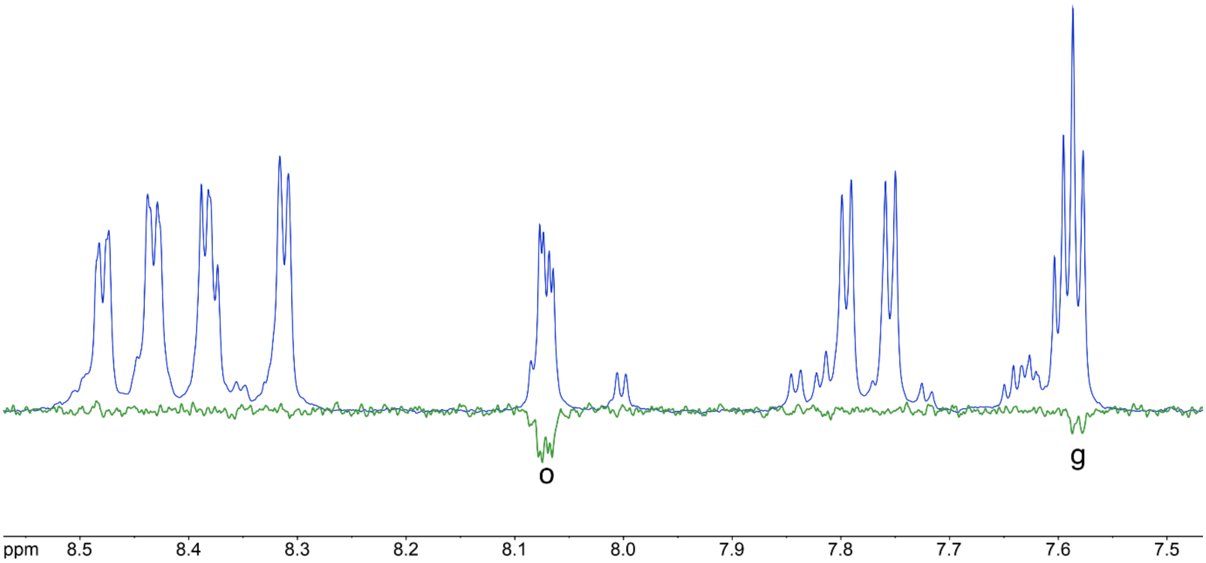

*Figure S16. The comparison of the aromatic region of the ^{1}H spectrum of 10^{-5} M dihexylquaterrylene (blue) and a 1D NOESY experiment, where the proton **f** was selectively excited, showing correlations to aromatic protons **o** and **g** (green). The mixing time was 0.5 s.*

Figure S16 shows the interaction between H**f** (aliphatic CH_2 group next to the aromatic ring) and the ring protons H**g** and H**o**. Some of the integrals of the peaks are shown in Figure S17 and S18 for the aliphatic and aromatic regions respectively. Since the minor isomer´s methyl group is in overlap with the major isomer signals, it is not possible to integrate them separately. The assignment of the peaks which are not integrated are not shown. The integral of peak **o** is close to one only when both of doublets are integrated which also applies for the methyl groups. This also points out to them being in the same isomer rather than belonging to different regioisomers. A very rough estimation of the ratio of the minor and major isomer is also possible to make, showing that the minor regiosomer is present in up to 15% amount. The chemical shifts and proton/carbon spectral assignment for 1,1'-dihexylquaterrylene are presented in Table S2.

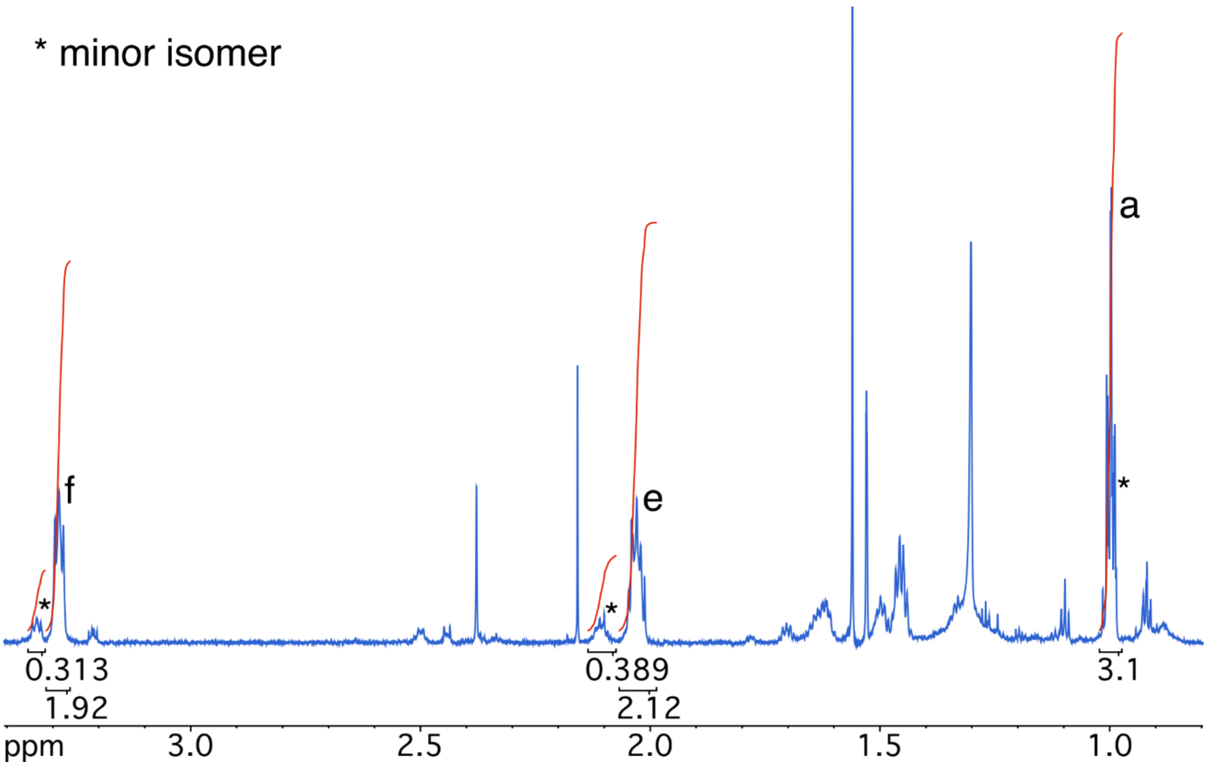

Figure S17. The integrals of certain peaks in the aliphatic region where the integration is possible.

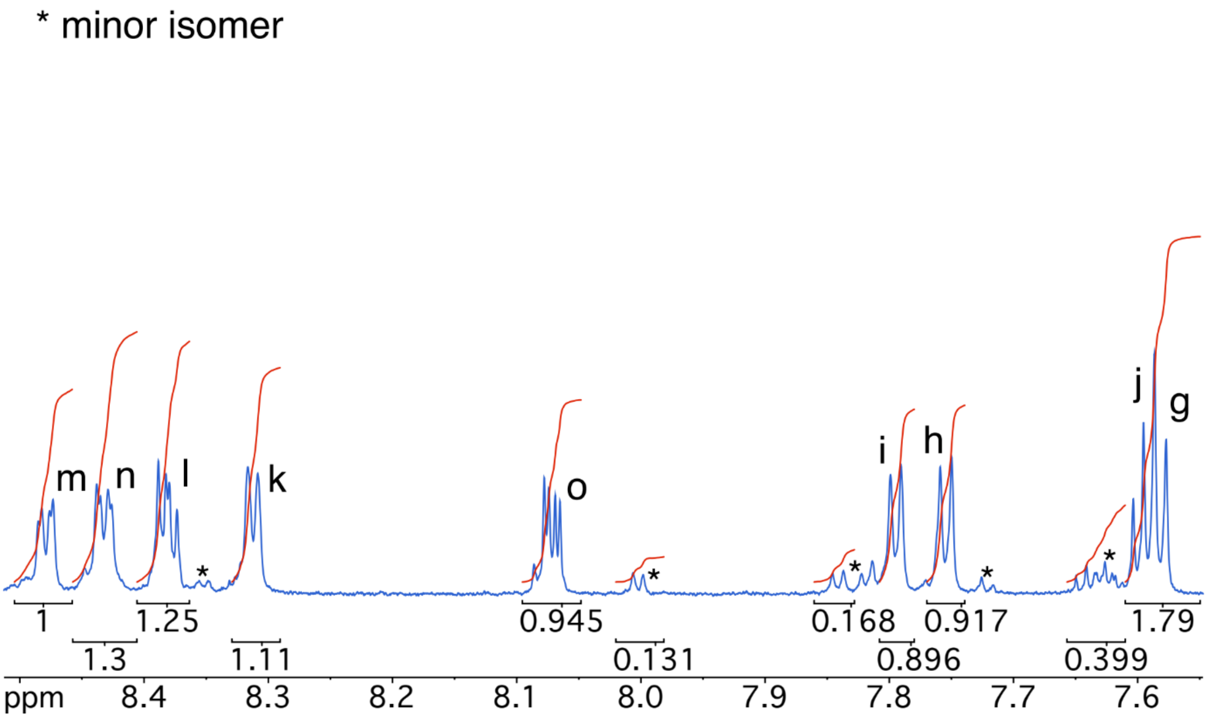

Figure S18. The integrals of peaks in the aromatic region.

Table S2. The spectral assignment of 1,1'-dihexylquaterrylene at 25 °C in CD_2Cl_2. The spectra have been recorded at 900MHz.

Site	$^1H/^{13}C$ Chemical shift in ppm	Note (Couplings and appearance)
Ha/Ca	0.96 / 14.26	t (appears: dt, 7.4 Hz)
Hb/Cb	1.41 / 23.17	s (appears: s 7.4Hz)
Hc/Cc	1.46 / 32.2	q (appears: q 6.6Hz)
Hd/Cd	1.58 / 30.00	multiplet
He/Ce	1.96 / 31.54	mutiplet
Hf/Cf	3.26 / 36.70	multiplet
Hg/Cg	7.53 / 131.39	d (appears: d, 8.3 Hz)
Hh/Ch	7.71 / 127.46	d (appears: d, 8.3 Hz)
Hi/Ci	7.75 / 127.74	d (appears: d, 7.8 Hz to j)
Hj/Cj	7.55 / 126.32	t (appears: t, 7.8 Hz to i, 7.3 Hz k)
Hk/Ck	8.27 / 121.28	d (appears: d, 7.3Hz to k)
Hl/Cl	8.34 / 121.71	d (appears: dd, 8.2 Hz)
Hm/Cm	8.44 / 121.62	d (appears: dd, 8.2Hz)
Hn/Cn	8.39 / 121.05	d (appears: dd, 7.9Hz)
Ho/Co	8.03 / 128.1	d (appears: dd, 7.9Hz)

4. Photophysical data

In order to verify that no non-linear processes affected the emission lifetime measurements of the J-aggregate, the fluorescence lifetime were measured at various excitation intensities. From Figure 19 it can be seen that the fluorescence lifetime is independent on excitation intensity, and hence any effect of non-linear processes or multi-excitation of the J-aggregates can be excluded at these low pulse energies (< 1.2 nJ/pulse).

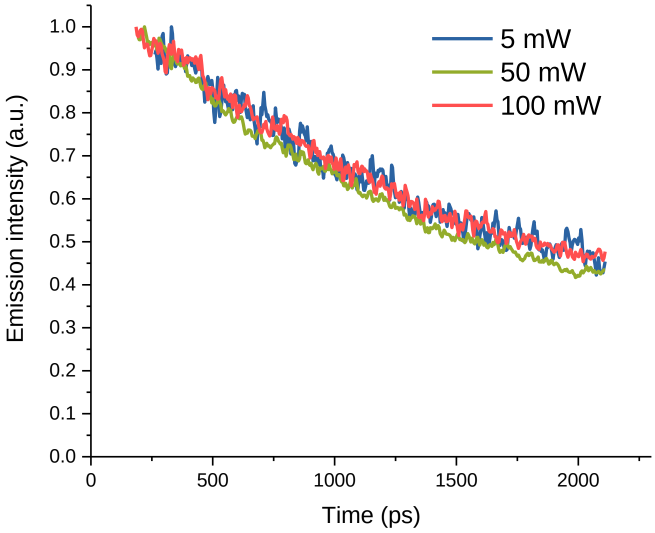

Figure 19. Normalized emission decay of J-aggregate at different excitation intensities. Measured using streak camera detection. Excitation wavelength 800 nm.

Table S3. Values for the energy difference between S_1 and S_0, the energy separation between a promoting vibrational mode, and the reorganization energy for both the Monomer and J-aggregate.

	ΔE (cm^{-1})	ω_{vib} (cm^{-1})	λ (cm^{-1})
Monomer	14947	1404	589
J-Aggregates	11974	1343	131

5. Diffusion NMR and diffusion coefficient simulation

Experimental diffusion coefficients

The diffusion NMR experiments were conducted in order to estimate the physical size of the aggregates. It should be noted that the NMR experiments were performed at 10^{-5} M and 10^{-4} M solutions, which is at the limit of sensitivity of the instrument for a reasonable timeframe of analysis. Thus, results presented below may provide an over-estimation of the physical size of 1,1'-dihexylquaterrylene J-aggregates. The diffusion coefficient for 1-hexylperylene could be determined even at concentrations >10^{-3} M in CD_2Cl_2 and $C_2D_2Cl_4$ since no signs of aggregation were observed. However, the diffusion coefficient for the 1,1'-dihexylquaterrylene monomer had to be determined at 10^{-5} M in CD_2Cl_2 since concentrations above that induce aggregation (Fig. 1). It should be noted that at just one order of magnitude higher concentration (10^{-4} M in CD_2Cl_2), 1,1'-dihexylquaterrylene seems to form aggregates that are in dynamic exchange with monomers. However, by using *ledbpgp2s*[1] and PROJECTED[2] sequences it was possible to suppress the exchange and therefore identify two separate diffusion coefficients that would allow to distinguish between the faster diffusing units (monomers) and the slowly diffusing units (aggregates). In case of $C_2D_2Cl_4$ the PROJECTED sequence was used to check that the diffusion coefficient of the monomer is the same no matter what sequence is used. Noteworthy, at both 10^{-5} M and 10^{-4} M in CD_2Cl_2 the diffusion coefficient for the monomeric specie is the same within the error of the measurement. This confirms that the exchange between the aggregate and the monomer is slow enough that it is possible to measure the separate diffusion coefficients for the monomer and for the aggregate in the same sample (Fig. S20-21). The experimental diffusion coefficients for 1-hexylperylene and 1,1'-dihexylquaterrylene at different concentrations are shown in the Table S4.

Table S4. The diffusion coefficient for the different compounds determined at 25 °C. All values were obtained using the ledbpgp2s and/or PROJECTED pulse sequence.

**Solvent**	CD_2Cl_2 [m^2/s]		$C_2D_2Cl_4$ [m^2/s]	
**Compound**	10^{-5} M	>10^{-5} M	10^{-4} M	>10^{-4} M
**1-hexylperylene**	-	$1.5 \cdot 10^{-9}$	-	$3.6 \cdot 10^{-10} \pm 6 \cdot 10^{-12}$
**QTRLN Monomer**	$1 \cdot 10^{-9} \pm 7 \cdot 10^{-11}$	$1 \cdot 10^{-9} \pm 3 \cdot 10^{-11}$	$2.6 \cdot 10^{-10} \pm 2 \cdot 10^{-12}$	$2.2 \cdot 10^{-10} \pm 1 \cdot 10^{-11}$
**QTRLN Aggregate**	-	$2.5 \cdot 10^{-10} \pm 3 \cdot 10^{-11}$	$6 \cdot 10^{-11} \pm 6 \cdot 10^{-12}$	$3.3 \cdot 10^{-11} \pm 1 \cdot 10^{-12}$

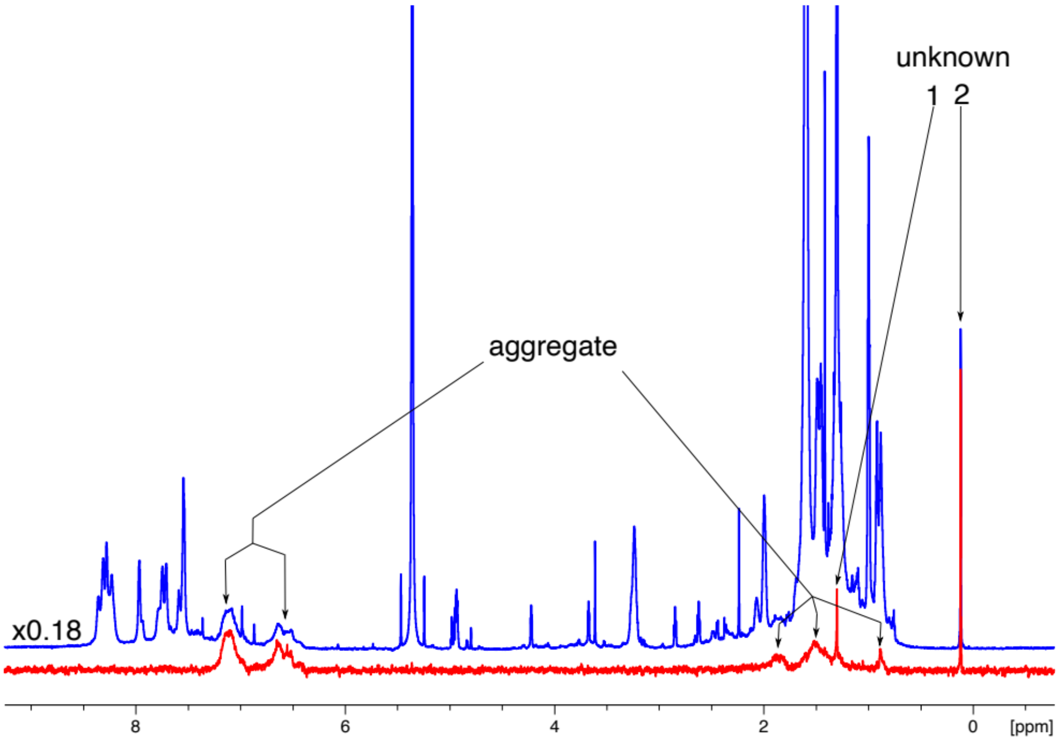

Figure S20. The 1st (blue spectrum) and 12th (red spectrum) slices of the pseudo 2D DOSY spectrum of 10^{-4} M 1,1'-dihexylquaterrylene in CD_2Cl_2. The gradient strength in the blue spectrum is 0.98 and in the red 34.44 Gauss/cm while the little delta was 4 ms and the big delta 50 ms.

In the diffusion coefficient measurement (DOSY) only the peak(s) around 7.1 ppm can be used for integration. The other peak(s) have either overlap in the first gradient value of the DOSY spectrum or have too low signal to noise and hence do not provide the desired fit quality. This can be clearly seen in Figure S22 where two slices of DOSY experiments are shown. The red line is at a higher gradient value (34.44 Gauss/cm) hence the fast diffusing species are filtered out including the dihexylquaterrylene monomer unit.

The aggregation pattern is even more prominent in $C_2D_2Cl_4$. The dynamic exchange seems to be present in this case as well as can be seen in Figure S23 where first and 12th slice of the DOSY pseudo 2D experiment is shown. As before, at the 12th slice where the gradient strength is 34.44 Gauss/cm only the slow diffusing components are present.

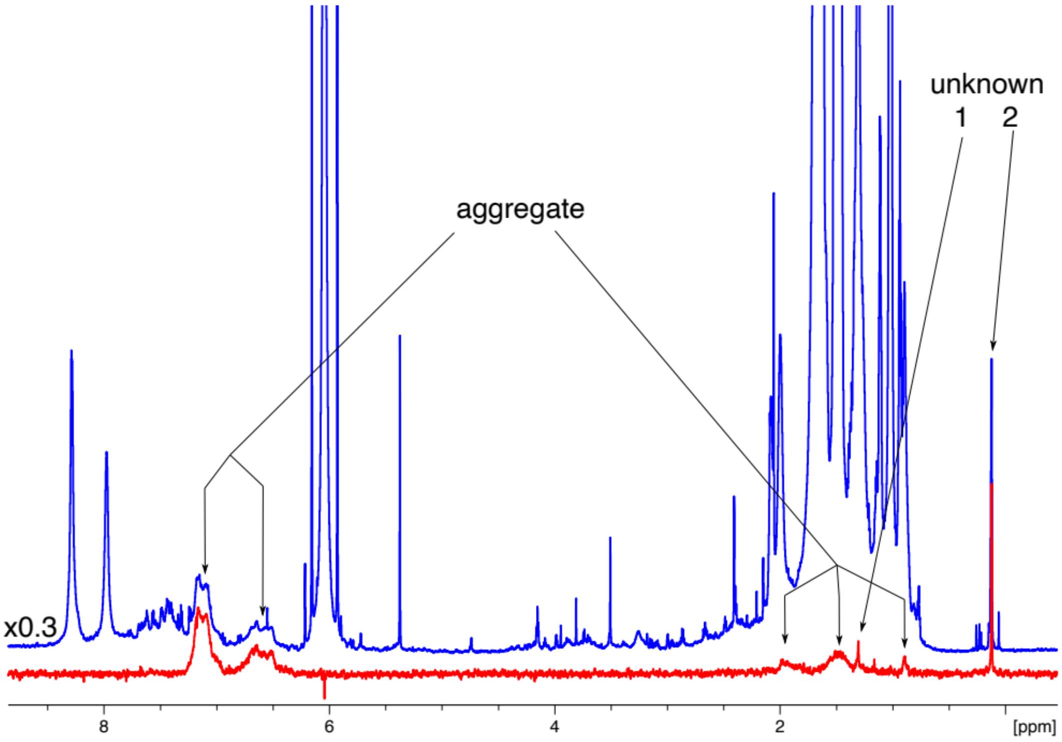

Figure S21. The 1st (blue spectrum) and 12th (red spectrum) slices of the pseudo 2D DOSY spectrum in high concentration $C_2D_2Cl_4$. The gradient strength in the blue spectrum is 0.98 and in the red 34.44 Gauss/cm while the little delta was 9.2ms and the big delta 60 ms.

The measurement of the diffusion coefficient was carried out using the exchange suppressed pulse sequence (PROJECTED[2]). This sequence requires that the transverse relaxation rates of the peaks in question is slow enough to survive the CPMG train. Only the methyl peak fulfils this requirement in at this concentration. The diffusion experiment shows that at larger concentrations (>10^{-3} M in $C_2D_2Cl_4$) 1,1-dihexylquaterrylene forms larger aggregates than at lower concentrations (10^{-4} M in $C_2D_2Cl_4$) because the diffusion rate is slower. The measurement error was assessed by variance reduction method and are presented in figures S22-23.

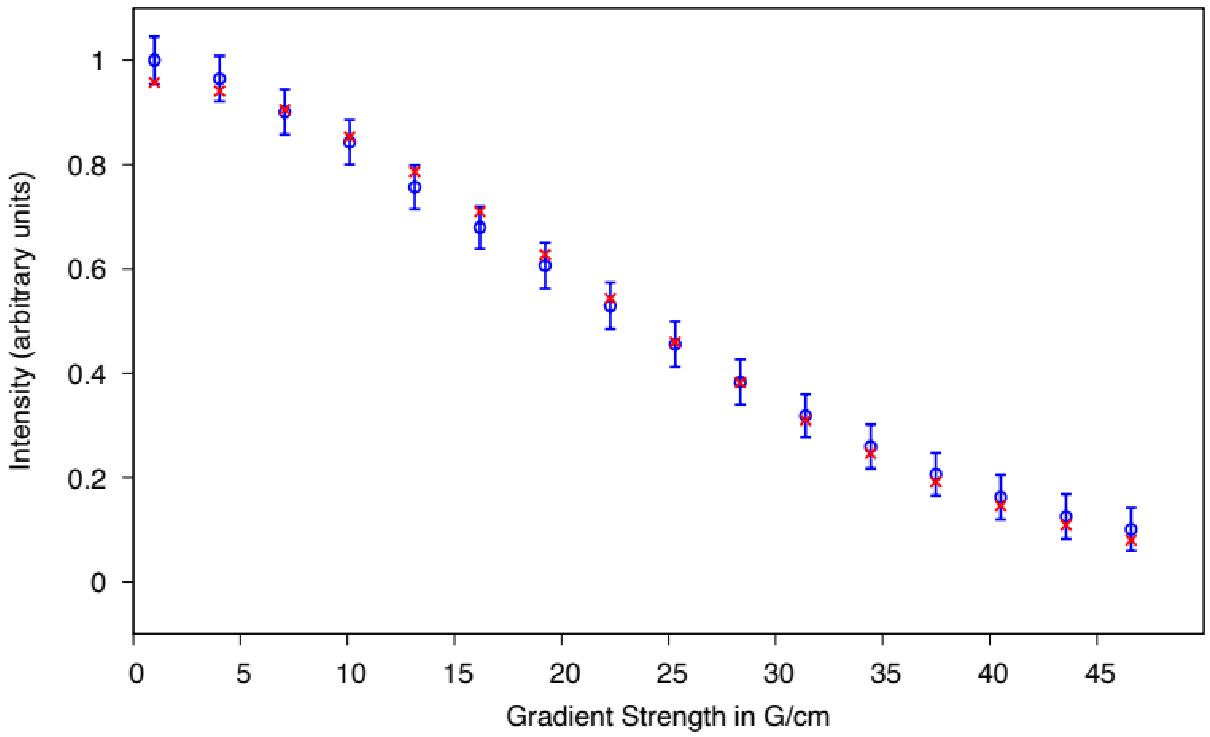

Figure S22.The diffusion decay curve for the J-aggregate in $C_2D_2Cl_4$ measured at 800 MHz and 25 °C. The blue circles with error bars denote the integrals and the red crosses denote the fitted points. The integral was taken between 7.2834 and 6.8817 ppm (centre at 7.0825 ppm). The variance reduction of the fit is 99.56.

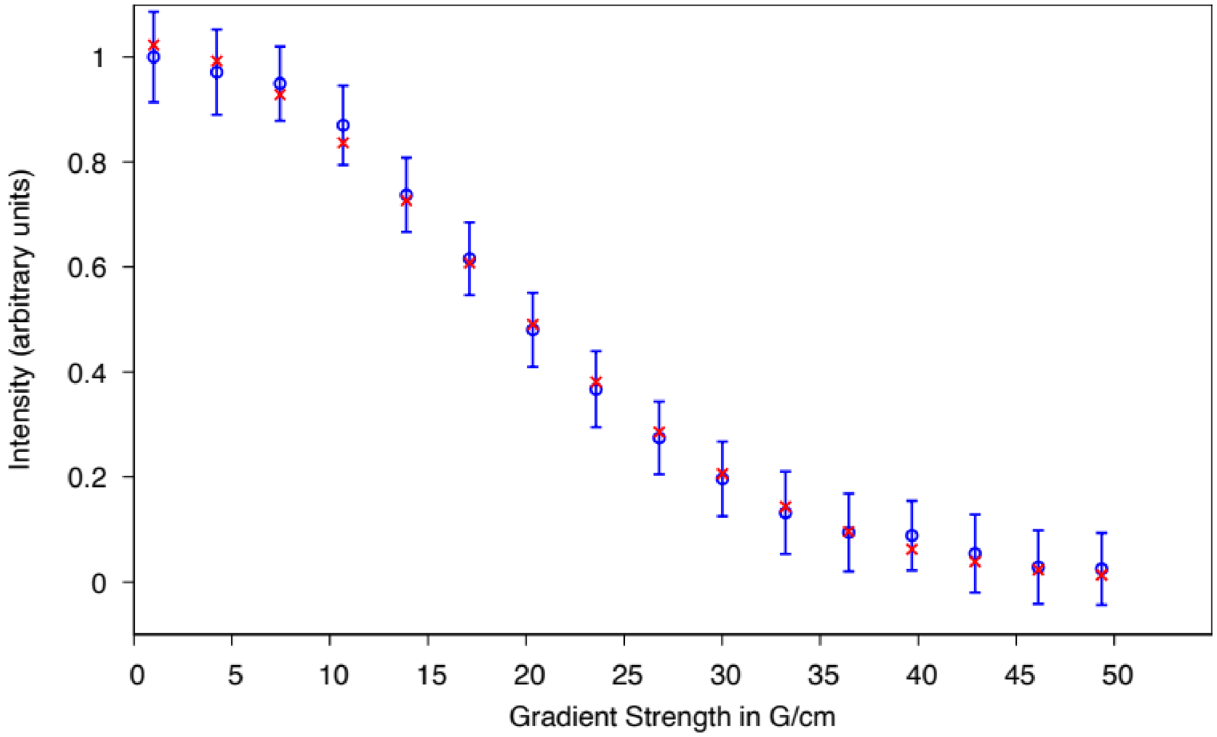

Figure S23. The DOSY decay curve for the aggregate in 10^{-4} M $C_2D_2Cl_4$ at 25 °C measured at 600 MHz with a high temperature curve. The peak around 7 ppm was integrated. The blue circles with error bars denote the integrals and the red crosses denote the fitted points. The variance reduction of the fitting is 99.8 revealing a good fit to data. Monte Carlo error estimation reveals an error connected to large signal to noise.

Simulated diffusion coefficients

The simulation of diffusion coefficients were performed using the HYDRO++10 software. The advantage of such simulation is that the software accounts for the ellipsoidal geometry of the constructed aggregate, whereas the Einstein-Stokes equation can only describe objects that can be approximated as spheres. The structure of the quaterrylene was optimized using MMFF94 force field from Open Babel within the Avogadro software. A simple python script was written to remove hydrogen from the xyz files and add a line to all the carbon atoms describing the radius of the sphere replacing the carbon atom in the HYDRO++10 simulation. A value of 0.7 Å was applied to all the carbons[4-5].

The diffusion coefficients were simulated first for the compounds where the structure is known. These were 1-hexylperylene and 1,1'-dihexylquaterrylene (monomer). The experimental and simulated diffusion constants are shown in Table S5. As seen from the table, simulated results show very good agreement with experimental results, thus this model was applied to estimate the physical size of the aggregates in CD_2Cl_2 and $C_2D_2Cl_4$. Larger complexes consisting of several units were built (manually without any type of quantum mechanical calculation as to geometry optimize the structure) and diffusion constants were simulated. Since J-aggregates typically occur in slip-stacked arrangement, the units were stacked on top of each other with a shift of roughly half of the molecular length. Distance between units was set at 3.5 Å, the distance between bay-substituted perylenes in π-π stacked complexes.[16] Simulated diffusion coefficients for aggregates containing up to 60 dihexylquaterrylene units are shown in Figure S26 and aggregation sizes matching the experimentally obtained diffusion coefficients are stated in Table S5.

Table S5. Experimental and HYDRO++10 simulated diffusion coefficients.

**Solvent** / **Compound**	CD_2Cl_2 [m²/s] experimental	CD_2Cl_2 [m²/s] simulated	$C_2D_2Cl_4$ [m²/s] experimental	$C_2D_2Cl_4$ [m²/s] simulated
**1-hexylperylene**	$1.5 \cdot 10^{-9} \pm 6 \cdot 10^{-12}$	$1.5 \cdot 10^{-9}$ / 1 unit	$3.6 \cdot 10^{-10} \pm 6 \cdot 10^{-12}$	$3.8 \cdot 10^{-10}$ / 1 unit
**1,1'-dihexylquaterrylene**	$1 \cdot 10^{-9} \pm 7 \cdot 10^{-11}$ [a]	$1 \cdot 10^{-9}$ / 1 unit	$6 \cdot 10^{-11} \pm 6 \cdot 10^{-12}$ [c]	$6.4 \cdot 10^{-11}$ / 14 units
	$1 \cdot 10^{-9} \pm 3 \cdot 10^{-11}$ [b]	$1 \cdot 10^{-9}$ / 1 unit	$3.3 \cdot 10^{-11} \pm 6 \cdot 10^{-12}$ [d]	$3.1 \cdot 10^{-11}$ / 34 units

[a] The diffusion constant was determined for 10^{-5} M sample in CD_2Cl_2, [b] The diffusion was determined for 10^{-4} M sample in CD_2Cl_2, [c] The diffusion constant was determined for 10^{-4} M sample in $C_2D_2Cl_4$, [d] The diffusion constant was determined for >10^{-3} M sample in $C_2D_2Cl_4$.

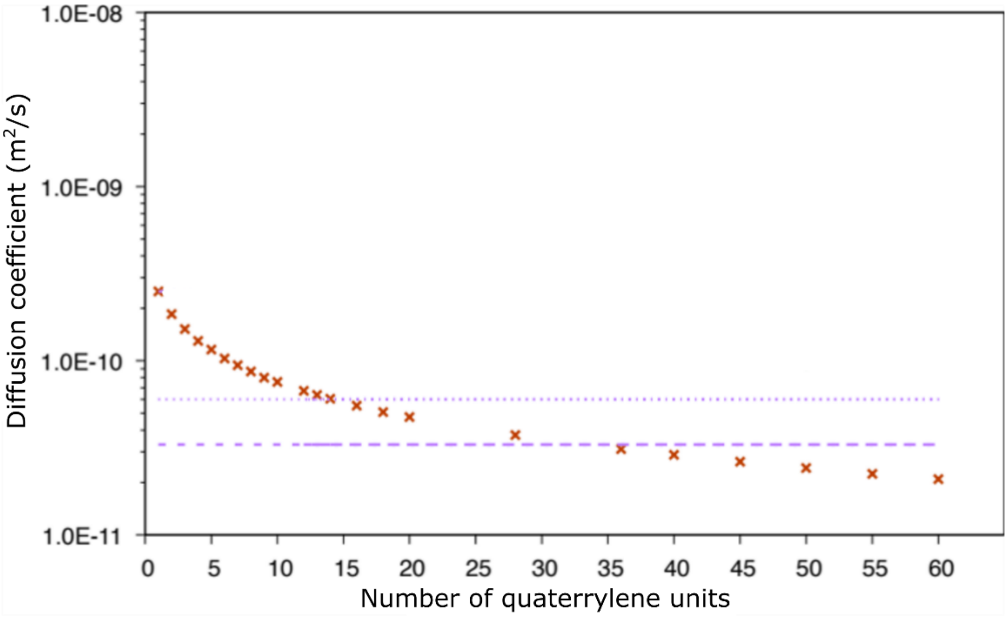

Figure S24. The simulated diffusion coefficients for aggregates built up by a maximum of 60 quaterrylene units. The orange crosses show the simulation in $C_2D_2Cl_4$. The dotted purple line represents the measured diffusion coefficient at 10^{-4} M $C_2D_2Cl_4$ and the dashed purple line at $>10^{-4}$ M $C_2D_2Cl_4$.

6. AFM and SEM images

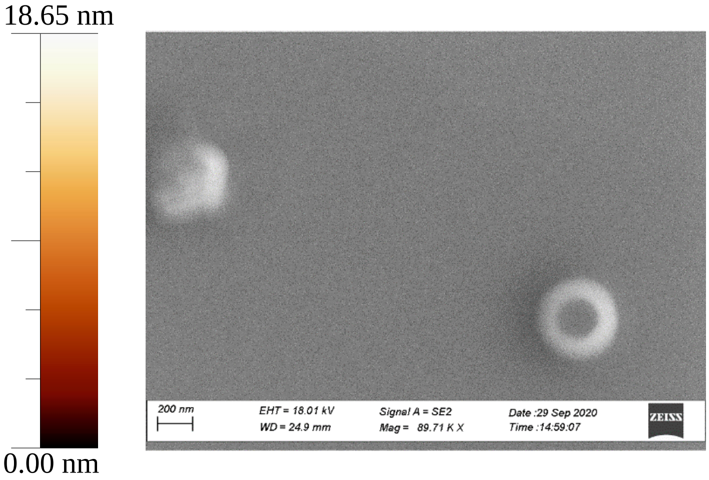

Figure S25. AFM (left) and SEM (right) images of an evaporated J-aggregate solution performed by drop casting on a silicon wafer chip.

7. References

1. Würth, C.; Grabolle, M.; Pauli, J.; Spieles, M.; Resch-Genger, U., Relative and absolute determination of fluorescence quantum yields of transparent samples. *Nature Protocols* **2013,** *8* (8), 1535-1550.

2. Rurack, K.; Spieles, M., Fluorescence Quantum Yields of a Series of Red and Near-Infrared Dyes Emitting at 600−1000 nm. *Analytical Chemistry* **2011,** *83* (4), 1232-1242.

3. Wu, D. H.; Chen, A. D.; Johnson, C. S., An Improved Diffusion-Ordered Spectroscopy Experiment Incorporating Bipolar-Gradient Pulses. *Journal of Magnetic Resonance, Series A* **1995,** *115* (2), 260-264.

4. Aguilar, J. A.; Adams, R. W.; Nilsson, M.; Morris, G. A., Suppressing exchange effects in diffusion-ordered NMR spectroscopy. *Journal of Magnetic Resonance* **2014,** *238*, 16-19.

5. Wolberg, J. In *Data Analysis Using the Method of Least Squares: Extracting the Most Information from Experiments*, 2005.

6. Garcia de la Torre, J.; Navarro, S.; Lopez Martinez, M. C.; Diaz, F. G.; Lopez Cascales, J. J., HYDRO: a computer program for the prediction of hydrodynamic properties of macromolecules. *Biophysical Journal* **1994,** *67* (2), 530-531.

7. García de la Torre, J.; del Rio Echenique, G.; Ortega, A., Improved Calculation of Rotational Diffusion and Intrinsic Viscosity of Bead Models for Macromolecules and Nanoparticles. *The Journal of Physical Chemistry B* **2007,** *111* (5), 955-961.

8. García de la Torre, J.; Carrasco, B., Hydrodynamic properties of rigid macromolecules composed of ellipsoidal and cylindrical subunits. *Biopolymers* **2002,** *63* (3), 163-167.

9. Ortega, A.; Amorós, D.; García de la Torre, J., Prediction of hydrodynamic and other solution properties of rigid proteins from atomic- and residue-level models. *Biophysical journal* **2011,** *101* (4), 892-898.

10. Acevedo, I. L.; Katz, M., Viscosities and thermodynamics of viscous flow of some binary mixtures at different temperatures. *Journal of Solution Chemistry* **1990,** *19* (10), 1041-1052.

11. Phillips, J. W. C.; Mumford, S. A., CCXXXII.—The dimorphism of certain higher aliphatic compounds. *Journal of the Chemical Society (Resumed)* **1931,** (0), 1732-1737.

12. Kushwaha, K.; Yu, L.; Stranius, K.; Singh, S. K.; Hultmark, S.; Iqbal, M. N.; Eriksson, L.; Johnston, E.; Erhart, P.; Müller, C.; Börjesson, K., A Record Chromophore Density in High-Entropy Liquids of Two Low-Melting Perylenes: A New Strategy for Liquid Chromophores. *Advanced Science* **2019,** *6* (4), 1801650.

13. Pell, A. J.; Edden, R. A. E.; Keeler, J., Broadband proton-decoupled proton spectra. *Magnetic Resonance in Chemistry* **2007,** *45* (4), 296-316.

14. Furrer, J., A robust, sensitive, and versatile HMBC experiment for rapid structure elucidation by NMR: IMPACT-HMBC. *Chemical Communications* **2010,** *46* (19), 3396-3398.

15. Kupce, E.; Freeman, R., Techniques for Multisite Excitation. *Journal of Magnetic Resonance, Series A* **1993,** *105* (2), 234-238.

16. Ye, C.; Gray, V.; Kushwaha, K.; Kumar Singh, S.; Erhart, P.; Börjesson, K., Optimizing photon upconversion by decoupling excimer formation and triplet triplet annihilation. *Physical Chemistry Chemical Physics* **2020,** *22* (3), 1715-1720.
